# Quasi-Maximum Exponential Likelihood Estimation of Conditional Quantiles for GARCH Models Based on High-Frequency Augmented Data

**DOI:** 10.3390/e28030326

**Published:** 2026-03-13

**Authors:** Zhenming Zhang, Shishun Zhao, Jianhua Cheng, Anze Wang

**Affiliations:** School of Mathematics, Jilin University, Changchun 130012, China; zhangzhenming@jlu.edu.cn (Z.Z.); zhaoss@jlu.edu.cn (S.Z.); wangaz1023@mails.jlu.edu.cn (A.W.)

**Keywords:** heavy-tailed distribution, the GARCH model, quasi-maximum exponential likelihood estimation, conditional quantile

## Abstract

GARCH models play a fundamental role in modeling time-varying volatility in financial return series. In practice, financial returns are also well known to exhibit heavy-tailed distributions, which naturally motivates the use of quasi-maximum exponential likelihood estimation (QMELE) for accurately capturing tail behavior and risk measures such as Value-at-Risk. At the same time, the increasing availability of intraday high-frequency data has led to the development of high-frequency augmented GARCH models, which incorporate intraday information into conventional low-frequency volatility frameworks. By exploiting transaction-level data recorded at very fine time scales, these models are able to capture intraday volatility dynamics and market microstructure effects that are not reflected in standard low-frequency observations. Against this background, this paper studies conditional quantile estimation for high-frequency augmented GARCH models. We develop QMELE-based estimators for both model parameters and conditional quantiles, and construct an adjusted test statistic for assessing model adequacy. The asymptotic properties of the proposed estimators and test statistic are established, and their finite-sample performance is examined through extensive simulation studies. Empirical applications to three major stock indices demonstrate that augmenting GARCH models with high-frequency information leads to substantial improvements in conditional quantile estimation compared with traditional low-frequency approaches.

## 1. Introduction

The application of time series data is pervasive across numerous real-world scenarios, especially within the realms of economics and finance. To effectively grasp the volatile nature of these intricate datasets, scholars have put forward a multitude of statistical models. Among them, the ARCH model (Autoregressive Conditional Heteroskedasticity model) together with the Generalized Autoregressive Conditional Heteroskedasticity (GARCH) model represent the two most prominent model representatives.

It is a well-established fact that Bollerslev [1] introduced the GARCH model in 1986. Subsequently, these model categories have undergone in-depth exploration and have been extensively applied across various fields. Here, the standard GARCH(p,q) model is defined as follows:

(1) $$\left\{\begin{array}{c} x_{t}=\sqrt{h_{t}}\eta_{t},\\ h_{t}=\alpha_{0}+\sum_{i=1}^{q}\alpha_{i}x_{t-i}^{2}+\sum_{j=1}^{p}\beta_{j}h_{t-j}, \end{array}\right.$$

where $x_{t}$ denote the financial return of a certain asset on the *t*-th day, $\alpha_{0}>0$, $\alpha_{i}\geq 0$, i=1,…,q, $\beta_{j}\geq 0$, j=1,…,p; The sequence $\left\{\eta_{t}\right\}$ constitutes random variables that are independent and identically distributed, with mean zero and variance one. Furthermore, $\eta_{t}$ is independent of all prior observations $x_{s}$ for any t>s. Consequently, given the information set at time t−1, the return $x_{t}$ has a conditional expectation of 0 and a conditional variance of $h_{t}$.

When estimating model parameters, Visser [2] employs the quasi-maximum likelihood estimation (QMLE) method. According to the theoretical properties of QMLE, the asymptotic normality of the resulting estimator relies on the existence of the fourth moment of the innovation process $\eta_{t}$ in (1). However, this moment condition is often difficult to satisfy in empirical applications. To relax such stringent moment requirements, a widely adopted alternative is estimation based on least absolute deviations (LADE) and its associated quasi-maximum exponential likelihood estimation (QMELE). Peng and Yao [3] applies the LADE method to the GARCH model and shows that the asymptotic properties of the estimator depend only on the second-order moment o $\eta_{t}$. Li and Li [4] further investigates the QMELE method for the ARFIMA-GARCH model and establish its asymptotic theory. Since then, QMELE has attracted considerable attention and has been applied to a wide range of time series models. For instance, Zhu and Ling [5] investigates the global and local convergence properties of QMELE for the ARMA-GARCH and IGARCH model, while Zhu and Ling [6] extends the method to the DAR(p) model. Pan and Chen [7] applies QMELE to GARCH(1,1) models that are non-stationary. Incorporating high-frequency intraday data, Huang et al. [8] develops an M-estimation approach for the GJR-GARCH model. Subsequently, Wang et al. [9] examines the use of composite quantile regression for parameter estimation of GARCH models based on daily observations.

Driven by the growing demands of financial institutions and regulatory authorities, the quantile-based estimation approach introduced by Koenker and Bassett [10] has become increasingly important in quantitative finance and empirical investment analysis. Consequently, the development of quantile regression methods for GARCH models has emerged as a natural and compelling research direction. This line of research leads to the study of the conditional quantiles of the GARCH(p,q) process, which can be expressed, by definition, as

(2) $$\begin{array}{c} Q_{\tau }\left(x_{t}|F_{t-1}\right)=Q_{\tau ,\eta }\sqrt{\alpha_{0}+\sum_{i=1}^{q}\alpha_{i}x_{t-i}^{2}+\sum_{j=1}^{p}\beta_{j}h_{t-j}},\;0<\tau <1, \end{array}$$

where $Q_{\tau ,\eta }$ denotes the τ-th quantile of the innovation process $\eta_{t}$, and $F_{t-1}$ represents the information set available up to time t−1.

It is well recognized that the square-root specification in Equation (2) results in an objective function that is not differentiable, while the recursive structure of the volatility process $h_{t}$ further complicates both asymptotic analysis and numerical implementation. To address these difficulties, alternative models featuring linear specifications for $h_{t}$ have been explored, dating back to the seminal work of Taylor [11]. Within this framework, Koenker and Zhao [12] develops quantile regression methods for conditional quantile estimation in linear ARCH-type models. This line of research is subsequently extended by Xiao and Koenker [13], which proposes a two-stage methodology for linear GARCH models. Despite these advances, the study of quantile regression for the standard GARCH model defined by (1) and (2) remains of considerable importance, as these specifications are more widely adopted in both empirical applications and theoretical investigations. In this direction, Lee and Noh [14] proposes a quantile regression framework for GARCH models based on a reparameterization strategy combined with local linear approximation. Similarly, Zheng et al. [15] develops a hybrid quantile regression estimator by applying a simple transformation to the conditional quantile expression in (2). For further discussion of computational issues arising in quantile regression, readers are referred to Koenker and Park [16].

With the swift advancement of electronic trading technologies, intraday high-frequency financial data have become increasingly accessible. Such data contain rich information on market dynamics and offer substantial potential for improving volatility modeling. However, as noted by Andersen and Bollerslev [17], standard GARCH models are generally not well suited for directly modeling intraday returns, as they may fail to capture certain features specific to high-frequency data. Motivated by this observation, Visser [2] proposes a pioneering scaling framework that incorporates intraday high-frequency information into a daily GARCH model. In this approach, high-frequency intraday returns are first aggregated into a daily volatility proxy, which is then employed within the conventional daily GARCH specification. This strategy allows high-frequency information to enhance volatility estimation accuracy without modifying the parametric structure of the underlying GARCH model. Building on the work of Visser [2], a growing body of literature has examined how the use of high-frequency volatility measures can improve parameter estimation and model performance across various GARCH-type models, see, among many others [8,18,19,20]. In what follows, we refer to this class of approaches, which enhance low-frequency GARCH models by incorporating high-frequency information through volatility proxies, as high-frequency augmented models

Despite their advantages, GARCH models applied to high-frequency data face several substantial challenges. In particular, Zhang et al. [21] point out that high-frequency financial data are often contaminated by market microstructure noise, which can severely impair estimation accuracy and lead to biased inference. Consequently, when working with intraday high-frequency observations, it is of considerable practical importance to develop robust methods for conditional quantile estimation within the GARCH framework. Nevertheless, to the best of our knowledge, relatively few studies have focused on conditional quantile estimation for GARCH models incorporating high-frequency information. Wang et al. [9] proposes a composite quantile regression approach for parameter estimation in such settings. However, their method relies on a reparameterization of the standard GARCH specification, in which $\alpha_{0}$ in (1) is either fixed at 1 or treated as a normalization constant, thereby enabling the application of the approach of Lee and Noh [14]. Moreover, their framework continues to face difficulties arising from the square-root structure in the conditional quantile representation (2). The primary objective of our paper is to address these challenges by developing a high-frequency augmented GARCH framework for parameter estimation and conditional quantile inference. By incorporating intraday high-frequency information into a conventional low-frequency GARCH model through volatility proxies, the proposed framework captures market dynamics more effectively without altering the original parametric structure. In addition, motivated by the heavy-tailed nature of financial returns, we adopt quasi-maximum exponential likelihood estimation (QMELE) for inference, which provides a robust alternative to likelihood-based methods under weak moment conditions. This feature distinguishes our approach from that of Zhang et al. [22], which employs quasi-maximum likelihood estimation (QMLE) within a related high-frequency augmented setting.

The remainder of this paper is organized as follows. Section 2 presents the QMELE estimation procedure for conditional quantiles in high-frequency augmented GARCH models, and establishes the asymptotic properties of the proposed estimators. Section 3 develops a model checking technique tailored to the high-frequency augmented framework. Section 4 reports extensive Monte Carlo simulations to evaluate the finite-sample performance of the proposed approaches, and compares them with conventional low-frequency quantile estimation methods. Section 5 presents an empirical application illustrating the practical relevance of the methodology. Section 6 concludes the paper. All technical proofs and derivations are relegated to the Appendix A for clarity.

## 2. QMELE Estimation and Asymptotic Properties

In this section, we develop the QMELE method for conditional quantile estimation in high-frequency augmented GARCH models, and establishes the corresponding asymptotic properties.

### 2.1. QMELE Estimation

First, we briefly review the hybrid quantile regression approach for GARCH models proposed by Zheng et al. [15]. Then, we introduce the QMELE estimation strategy incorporating intraday high-frequency information, following the framework of Visser [2]. Within this setting, our objective is to integrate high-frequency intraday information into the hybrid quantile estimation framework, thereby improving the accuracy of parameter and conditional quantile estimation.

#### 2.1.1. Conditional Quantile Estimation via QMLE in GARCH Models

This section considers a strictly stationary and ergodic GARCH(p,q) process defined by model (1), which generates the random sample series $\left\{x_{t},\;t=1,\ldots ,n\right\}$. Let $F_{t}$ denote the σ-field generated by past observations $\left\{x_{t},x_{t-1},\ldots \right\}$. Following Zheng et al. [15], define the transformation $y_{t}=T\left(x_{t}\right)=x_{t}^{2}\mathrm{sgn}\left(x_{t}\right)$, and let $b_{\tau }=T\left(Q_{\tau ,\eta }\right)$, where $Q_{\tau ,\eta }$ denotes the τ-th quantile of the innovation $\eta_{t}$. Under this setting, the τ-th conditional quantile of the transformed series $y_{t}$, given the information set $F_{t-1}$, can be expressed as

$$\begin{array}{c} Q_{\tau }\left(y_{t}|F_{t-1}\right)=b_{\tau }\left(\alpha_{0}+\sum_{i=1}^{q}\alpha_{i}x_{t-i}^{2}+\sum_{j=1}^{p}\beta_{j}h_{t-j}\right)=\theta_{\tau }^{\prime }z_{t},\;0<\tau <1, \end{array}$$

where

(3) $$\begin{array}{c} z_{t}={\left(1,x_{t-1}^{2},\ldots ,x_{t-q}^{2},h_{t-1},\ldots ,h_{t-p}\right)}^{\prime },\;\;\theta_{\tau }=b_{\tau }{\left(\alpha_{0},\alpha_{1},\ldots ,\alpha_{q},\beta_{1},\ldots ,\beta_{p}\right)}^{\prime }. \end{array}$$

If the values of $\left\{h_{t}\right\}$ are observed, the conditional quantile $Q_{\tau }\left(y_{t}|F_{t-1}\right)$ can be estimated using a quantile regression framework with linear specification.

Let the parameter vector

$$\theta ={\left(\alpha_{0},\alpha_{1},\ldots ,\alpha_{q},\beta_{1},\ldots ,\beta_{p}\right)}^{\prime }$$

denote the vector collecting all coefficients to be estimated in the model. To implement the two-stage hybrid estimation procedure, we impose the following conditions: there exist constants $\rho_{0}$, $\rho_{1}$ and $\rho_{2}$ such that $0<\rho_{1}<\rho_{2}$, $0<\rho_{0}<1$ and $p\rho_{1}<\rho_{0}$. Under these conditions, the parameter space is defined as

$$\begin{array}{cc} \Theta =\left\{\theta :\beta_{1}+\cdots +\beta_{p}⩽\rho_{0},\rho_{1}\right. & ⩽\min\left(\alpha_{0},\alpha_{1},\ldots ,\alpha_{q},\beta_{1},\ldots ,\beta_{p}\right)\\ & \left.⩽\max\left(\alpha_{0},\alpha_{1},\ldots ,\alpha_{q},\beta_{1},\ldots ,\beta_{p}\right)⩽\rho_{2}\right\}\subset R_{+}^{p+q+1}, \end{array}$$

where $R_{+}=\left(0,\infty \right)$. Compared with the standard non-negativity conditions

$$\alpha_{0}>0,\;\alpha_{i}⩾0,\;1⩽i⩽q,\;\beta_{j}⩾0,\;1⩽j⩽p,$$

the parameter space Θ is slightly more restrictive, since it additionally imposes uniform lower and upper bounds on the parameters and an upper bound on $\beta_{1}+\ldots +\beta_{p}$. Throughout the paper, we assume that the true parameter θ lies in the interior of Θ.

We denote the true value of the model parameters by

$$\theta_{0}={\left(\alpha_{00},\alpha_{01},\ldots ,\alpha_{0q},\beta_{01},\ldots ,\beta_{0p}\right)}^{\prime },$$

then we specify $h_{t}\left(\theta \right)$ recursively as

(4) $$\begin{array}{c} h_{t}\left(\theta \right)=\alpha_{0}+\sum_{i=1}^{q}\alpha_{i}x_{t-i}^{2}+\sum_{j=1}^{p}\beta_{j}h_{t-j}\left(\theta \right). \end{array}$$

For convenience, we write $h_{t}\left(\theta_{0}\right)=h_{t}$. Observe that the recursive computation of $h_{t}\left(\theta \right)$ requires initial values for $\left\{x_{0}^{2},\ldots ,x_{1-q}^{2},h_{0},\ldots ,h_{1-p}\right\}$. In this study, these initial values are set equal to the sample mean $n^{-1}\sum_{t=1}^{n}x_{t}^{2}$, then the resulting sequence is denoted by ${\tilde{h}}_{t}\left(\theta \right)$. The main steps of the conditional quantile estimation procedure proposed by Zheng et al. [15] are summarized as follows.

- Step E1 (QMLE Estimation for GARCH parameters): Utilize QMLE to estimate the parameters for the GARCH(p,q) model, i.e.,
  (5) $$\begin{array}{c} {\tilde{\theta }}_{n}=\underset{\theta \in \Theta }{\mathrm{argmin}}\sum_{t=1}^{n}{\tilde{\ell }}_{t}\left(\theta \right), \end{array}$$
  with ${\tilde{\ell }}_{t}\left(\theta \right)=\log\left({\tilde{h}}_{t}\left(\theta \right)\right)+x_{t}^{2}/{\tilde{h}}_{t}\left(\theta \right)$, which defined in Francq and Zakoian [23]. Consequently, we compute the estimated values $\left\{h_{t}\right\}$ as ${\tilde{h}}_{t}={\tilde{h}}_{t}\left({\tilde{\theta }}_{n}\right)$.
- Step E2 (Conditional Quantile Regression) Define the regressor vector
  $${\tilde{z}}_{t}={\left(1,x_{t-1}^{2},\ldots ,x_{t-q}^{2},{\tilde{h}}_{t-1},\ldots ,{\tilde{h}}_{t-p}\right)}^{\prime }.$$
  Using the estimated conditional variances $\left\{\tilde{h_{t}}\right\}$ as weights, the τ-th conditional quantile regression of the transformed series $\left\{y_{t}\right\}$ is obtained by solving the following weighted quantile estimation problem:
  $$\begin{array}{c} {\hat{\theta }}_{\tau n}=\underset{\theta_{\tau }}{\mathrm{argmin}}\sum_{t=1}^{n}\frac{1}{{\tilde{h}}_{t}}\rho_{\tau }\left(y_{t}-\theta_{\tau }^{\prime }{\tilde{z}}_{t}\right), \end{array}$$
  where the check loss function is defined as $\rho_{\tau }\left(x\right)=x\left(\tau -I\left\{x<0\right\}\right)$ for a fixed τ∈(0,1). Accordingly, the τ-th quantile of $\left\{y_{t}\right\}$ given $F_{t-1}$ is estimated by
  $${\hat{Q}}_{\tau }\left(y_{t}|F_{t-1}\right)={\hat{\theta }}_{\tau n}^{\prime }{\tilde{z}}_{t}.$$
- Step E3 (Transformation to the Original Model) The τ-th conditional quantile of the original return series $\left\{x_{t}\right\}$ is obtained by applying the inverse transformation
  $${\hat{Q}}_{\tau }\left(x_{t}|F_{t-1}\right)=T^{-1}\left({\hat{\theta }}_{\tau n}^{\prime }{\tilde{z}}_{t}\right),$$
  where $T^{-1}\left(x\right)=$$\sqrt{\left|x\right|}\mathrm{sgn}\left(x\right)$.

#### 2.1.2. QMELE Estimation for High-Frequency Augmented GARCH Models

Let $\theta ={\left(\alpha_{0},\alpha_{1},\ldots ,\beta_{p}\right)}^{\prime }$ denote the vector of unknown parameters to be estimated, and let $X_{t}\left(u\right)$ represent the intraday log-return of a given asset observed at time *u* on day *t*. Following Visser [2], the daily trading period is normalized to the unit interval [0,1], which leads to the following scaled model:

(6) $$\left\{\begin{array}{c} X_{t}\left(u\right)=\sqrt{h_{t}}Z_{t}\left(u\right),\;0⩽u⩽1,\\ h_{t}=\alpha_{0}+\sum_{i=1}^{q}\alpha_{i}x_{t-i}^{2}+\sum_{j=1}^{p}\beta_{j}h_{t-j}, \end{array}\right.$$

in which $h_{t}$ indicates the daily volatility level. Under the QMELE framework, the standardized intraday innovations $Z_{t}\left(u\right)$ are assumed to be independent and identically distributed across trading days. Their sample paths are right-continuous with left limits and satisfy the normalization condition $E(|Z_{t}\left(1\right)|)=1$. The specification in the above equation explicitly incorporates intraday high-frequency observation $X_{t}\left(u\right)$ into the daily volatility dynamics. Moreover, it reduces to the conventional GARCH model in Equation (1) by setting

$$x_{t}\equiv X_{t}\left(1\right),\;\;\eta_{t}\equiv Z_{t}\left(1\right).$$

For estimation convenience, it is necessary to construct volatility proxies that effectively summarize information contained in intraday price movements. A key property shared by these proxies is positive homogeneity. Specifically, for the intraday return process $X_{t}\left(u\right)$ and any positive constant ρ>0, the following relationship holds:

$$\begin{array}{c} H\left(\rho X_{t}\left(u\right)\right)=\rho H\left(X_{t}\left(u\right)\right)>0, \end{array}$$

where H(·) denotes a so-called volatility proxy defined as a positive functional of the intraday high-frequency data. Because the volatility level $h_{t}$ is constant over each trading day, the property of positive homogeneity leads to the following result:

$$\begin{array}{c} H_{t}\equiv H\left(X_{t}\left(u\right)\right)=H\left(\sqrt{h_{t}}Z_{t}\left(u\right)\right)=\sqrt{h_{t}}H\left(Z_{t}\left(u\right)\right). \end{array}$$

Define

$$\begin{array}{c} z_{H,t}=H\left(Z_{t}\left(u\right)\right),\;\mu =E\left(z_{H,t}\right),\;\varepsilon_{t}^{⋆}=\frac{z_{H,t}}{\mu }, \end{array}$$

then we have $E(\varepsilon_{t}^{⋆})=1$, where $\left\{\varepsilon_{t}^{⋆}\right\}$ forms an i.i.d. sequence. This follows from the assumption that the standardized intraday process $\left\{Z_{t}\left(u\right)\right\}$ is i.i.d. across trading days. Accordingly, the volatility proxy model admits the following representation:

(7) $$\left\{\begin{array}{c} H_{t}=\sqrt{h_{t}}z_{H,t}=\sqrt{h_{t}}\mu \varepsilon_{t}^{⋆},\\ h_{t}=\alpha_{0}+\sum_{i=1}^{q}\alpha_{i}x_{t-i}^{2}+\sum_{j=1}^{p}\beta_{j}h_{t-j}. \end{array}\right.$$

Along with the primary parameter vector $\theta ={\left(\alpha_{0},\alpha_{1},\ldots ,\beta_{p}\right)}^{\prime }$, model (7) involves an additional scale parameter μ. Because this extra parameter leads to an identification issue, the QMELE approach is unable to estimate all parameters simultaneously. To circumvent this difficulty, we introduce the transformation $\sqrt{h_{t}^{⋆}}=\sqrt{h_{t}}\mu$, under which model (7) can be rewritten as follows:

(8) $$\left\{\begin{array}{c} H_{t}=\sqrt{h_{t}^{⋆}}\varepsilon_{t}^{⋆},\\ h_{t}^{⋆}=\alpha_{0}^{⋆}+\sum_{i=1}^{q}\alpha_{i}^{⋆}x_{t-i}^{2}+\sum_{j=1}^{p}\beta_{j}^{⋆}h_{t-j}^{⋆}, \end{array}\right.$$

in which

(9) $$\begin{array}{c} \alpha_{0}^{⋆}=\alpha_{0}\mu^{2},\;\;\alpha_{i}^{⋆}=\alpha_{i}\mu^{2},\;\;\beta_{j}^{⋆}=\beta_{j},\;\;i=1,2,\ldots ,q,\;j=1,2,\ldots ,p. \end{array}$$

Noting that we consider the special case $H_{t}=\left|X_{t}\left(1\right)\right|=\left|x_{t}\right|$, it follows that

$$\mu =1,\;\sqrt{h_{t}^{⋆}}=\sqrt{h_{t}},$$

This observation shows that model (8) nests the standard GARCH model (1) as a special case.

In line with Visser [2], let

$$\theta_{0}^{⋆}={\left(\alpha_{00}^{⋆},\alpha_{01}^{⋆},\ldots ,\alpha_{0q}^{⋆},\beta_{01}^{⋆},\ldots ,\beta_{0p}^{⋆}\right)}^{\prime }$$

denote the true parameter vector. For the transformed model (8), the Laplace-based quasi-maximum exponential likelihood is adopted to estimate $\theta^{⋆}={\left(\alpha_{0}^{⋆},\alpha_{1}^{⋆},\ldots ,\alpha_{q}^{⋆},\beta_{1}^{⋆},\ldots ,\beta_{p}^{⋆}\right)}^{\prime }$. Specifically, the QMELE estimator is defined as

(10) $$\begin{array}{c} {\hat{\theta }}^{⋆}=\underset{\theta^{⋆}\in \Theta^{⋆}}{\arg\min}\sum_{t=1}^{n}l_{t}\left(\theta^{⋆}\right), \end{array}$$

in which $\Theta^{⋆}$ is a compact subspace of the space restricted by $\alpha_{i}^{⋆}\geq 0$, i=0,1,…,q, $0\leq \sum_{j=1}^{q}\beta_{j}^{⋆}<1$, and the objective function is given by

$$\begin{array}{c} l_{t}\left(\theta^{⋆}\right)=\log\left(\sqrt{h_{t}^{⋆}\left(\theta^{⋆}\right)}\right)+\frac{|H_{t}|}{\sqrt{h_{t}^{⋆}\left(\theta^{⋆}\right)}}. \end{array}$$

Analogous to Visser [2], the resulting estimator satisfies the asymptotic normality

$$\begin{array}{c} \sqrt{n}\left({\hat{\theta }}^{⋆}-\theta_{0}^{⋆}\right)\overset{d}{⟶}N\left(0,\Sigma_{1}^{⋆}\right),\;\;n\to \infty , \end{array}$$

where $\Sigma_{1}^{⋆}=G^{⋆-1}I^{⋆}G^{⋆-1}$ with

$${\left(G^{⋆}\right)}_{i,j}=E\left(\frac{\partial^{2}l_{t}\left(\theta_{0}^{⋆}\right)}{\partial \theta_{i}^{⋆}\partial \theta_{j}^{⋆}}\right),\;\;{\left(I^{⋆}\right)}_{i,j}=E\left(\frac{\partial l_{t}\left(\theta_{0}^{⋆}\right)}{\partial \theta_{i}^{⋆}}\frac{\partial l_{t}\left(\theta_{0}^{⋆}\right)}{\partial \theta_{j}^{⋆}}\right).$$

Furthermore, once an estimator of μ is available (as presented in Section 4), the parameter θ can then be recovered according to (9).

#### 2.1.3. Conditional Quantile Estimation via QMELE in High-Frequency Augmented GARCH Models

Building on the preceding discussions, our primary objective is to improve conditional quantile estimation within the GARCH (p,q) framework, by exploiting intraday high-frequency information. To this end, we naturally adopt the QMELE approach based on the volatility proxy model defined in Equation (8), as introduced in Step E1 and detailed in Section 2.1.1. The estimation of economically relevant conditional quantiles is then carried out following the sequential procedures described in Step E2 and Step E3.

Below, we outline the proposed hybrid estimation procedure and its implementation steps.

- Step M1 (Preliminary QMELE Estimation) implement the QMELE procedure for the volatility proxy specification in (8), which incorporates intraday high-frequency information, to obtain a preliminary estimator of $\theta_{n}^{⋆}$. Specifically,
  (11) $$\begin{array}{c} {\tilde{\theta }}_{n}^{⋆}=\underset{\theta \in \Theta^{⋆}}{\mathrm{argmin}}\sum_{t=1}^{n}{\tilde{\ell }}_{t}^{⋆}\left(\theta \right), \end{array}$$
  where the objective function is defined as
  $${\tilde{\ell }}_{t}^{⋆}\left(\theta \right)=\log\left(\sqrt{{\tilde{h}}_{t}^{⋆}\left(\theta \right)}\right)+\frac{|H_{t}|}{\sqrt{{\tilde{h}}_{t}^{⋆}\left(\theta \right)}}.$$
  The recursive construction of ${\tilde{h}}_{t}^{⋆}\left(\theta \right)$ requires initial values $\left\{x_{0}^{2},\ldots ,x_{1-q}^{2},h_{0}^{⋆},\ldots ,h_{1-p}^{⋆}\right\}$, which are all set equal to the sample mean $n^{-1}\sum_{t=1}^{n}x_{t}^{2}$. Given ${\tilde{\theta }}_{n}^{⋆}$, the original parameter vector θ can be recovered according to (9) as ${\hat{\theta }}_{n}^{⋆\prime }={\tilde{\theta }}_{n}^{⋆\prime }\hat{M}$ where
  (12) $$\hat{M}=\mathrm{diag}\left\{\underset{q+1}{\underset{︸}{1/{\hat{\mu }}^{2},\ldots ,1/{\hat{\mu }}^{2}}},\underset{p}{\underset{︸}{1,\ldots ,1}}\right\},$$
  and $\hat{\mu }$ denotes an estimator of μ. Subsequently, the fitted conditional variances $\left\{h_{t}\right\}$ are computed as ${\tilde{h}}_{t}^{⋆}={\tilde{h}}_{t}^{⋆}\left({\hat{\theta }}_{n}^{⋆}\right)={\tilde{h}}_{t}\left({\hat{\theta }}_{n}^{⋆}\right)$. This step closely parallels the QMELE-based procedure proposed by Visser [2].
- Step M2 (Conditional Quantile Estimation with High-Frequency Augmented Data) Let
  $${\tilde{z}}_{t}^{⋆}={\left(1,x_{t-1}^{2},\ldots ,x_{t-q}^{2},{\tilde{h}}_{t-1}^{⋆},\ldots ,{\tilde{h}}_{t-p}^{⋆}\right)}^{\prime },$$
  using the fitted conditional variances $\left\{{\tilde{h}}_{t}\right\}$ as weights, the τ-th conditional quantile regression of the transformed process $\left\{y_{t}\right\}$ is obtained by solving
  $$\begin{array}{c} {\hat{\theta }}_{\tau n}^{⋆}=\underset{\theta_{\tau }}{\mathrm{argmin}}\sum_{t=1}^{n}\frac{1}{{\tilde{h}}_{t}^{⋆}}\rho_{\tau }\left(y_{t}-\theta_{\tau }^{\prime }{\tilde{z}}_{t}^{⋆}\right), \end{array}$$
  where $\rho_{\tau }\left(\cdot \right)$ denotes the check loss function. Accordingly, the τ-th quantile of $\left\{y_{t}\right\}$ given $F_{t-1}$ is estimated by
  $${\hat{Q}}_{\tau }^{⋆}\left(y_{t}|F_{t-1}\right)={\hat{\theta }}_{\tau n}^{⋆\prime }{\tilde{z}}_{t}^{⋆}.$$
- Step M3 (Conditional Quantile for the Daily Model) For the daily return series $\left\{x_{t}\right\}$, the τ-th conditional quantile implied by the GARCH model (1) is obtained through the inverse transformation
  $${\hat{Q}}_{\tau }^{⋆}\left(x_{t}|F_{t-1}\right)=T^{-1}\left({\hat{\theta }}_{\tau n}^{⋆\prime }{\tilde{z}}_{t}^{⋆}\right),$$
  where the inverse transformation is defined as
  $$T^{-1}\left(x\right)=\sqrt{\left|x\right|}\mathrm{sgn}\left(x\right).$$

The above procedure allows for the joint estimation of the parameter vector θ and the corresponding conditional quantiles by effectively combining low-frequency returns with intraday high-frequency information. The resulting conditional quantiles play a central role in quantile-based risk assessment, including widely used measures such as Value-at-Risk (VaR) and Expected Shortfall (ES). By incorporating high-frequency information into a unified modeling framework, the proposed approach exploits richer intraday dynamics and provides a more informative characterization of volatility behavior. Accordingly, we refer to the resulting methodology as high-frequency augmented conditional quantile estimation, which offers a flexible and refined representation of financial market volatility.

Next, building on the foregoing theoretical developments, we investigate the asymptotic properties of the proposed estimators obtained under the high-frequency augmented conditional quantile estimation framework. Our analysis shows that the appropriate incorporation of intraday high-frequency information in Step E1 substantially affects the asymptotic covariance structure of the estimators. This effect, in turn, leads to notable improvements in both estimation efficiency and predictive accuracy.

### 2.2. Asymptotic Properties

Before presenting the asymptotic results, we introduce the following assumptions.

**Assumption** **1.** *The polynomials $\sum_{i=1}^{q}\alpha_{i}^{⋆}x^{i}$ and $1-\sum_{j=1}^{p}\beta_{j}^{⋆}x^{j}$ have no common roots.*

**Assumption** **2.** *The innovation process satisfies $E(\eta_{t}^{2})=1$, and $\eta_{t}^{2}$ is non-degenerate.*

**Assumption** **3.** *$E\left[\log\left(\left(\sum_{i=1}^{q}\alpha_{0i}\right)\eta_{t}^{2}+\sum_{j=1}^{p}\beta_{0j}\right)\right]<0$.*

**Assumption** **4.** *The true parameter $\theta_{0}^{⋆}$ lies in the interior of the compact parameter space $\Theta^{⋆}$.*

**Assumption** **5.** *$E(\varepsilon_{t}^{⋆2})<\infty$.*

**Assumption** **6.** *The transformed innovation $\varepsilon_{t}=T\left(\eta_{t}\right)$ admits a density function f(·) that is positive and continuously differentiable, with derivative $\dot{f}\left(\cdot \right)$ satisfying $\sup_{x\in R}\left|\dot{f}\left(x\right)\right|<\infty$.*

Assumptions 2 and 3 are a sufficient conditions for ensuring ergodic and strict stationary of the GARCH model (1) according to [23]. Assumptions 1, 4 and 5 are imposed to establish the asymptotic normality and consistency of the QMELE estimator ${\tilde{\theta }}_{n}$. Notably, these conditions require only the existence of a bounded fractional moment of $x_{t}$, such as $E(|x_{t}{|}^{2\delta_{0}})<\infty$ for some $\delta_{0}>0$. This requirement substantially enlarges the admissible parameter space of the GARCH model, since higher-order moment assumptions are known to impose restrictive constraints on the parameter region. Finally, Assumption 6 is a standard regularity condition in quantile-based estimation and is introduced to facilitate the technical derivations.

Based on the notations $z_{H,t}=H\left(Z_{t}\left(u\right)\right)$ and $\varepsilon_{t}^{⋆}=z_{H,t}/\mu$, we define

$$\kappa_{1}^{⋆}=\frac{1}{2}\left(E\left[\varepsilon_{t}^{⋆}I\left(\eta_{t}<Q_{\tau ,\eta }\right)\right]-\tau \right),\;\kappa_{2}^{⋆}=4\mathrm{Var}\left[\varepsilon_{t}^{⋆}\right],$$

and furthermore define

$$\begin{array}{c} J^{⋆}=E\left[\frac{1}{h_{t}^{⋆2}\left(\theta_{0}^{⋆}\right)}\frac{\partial h_{t}^{⋆}\left(\theta_{0}^{⋆}\right)}{\partial \theta^{⋆}}\frac{\partial h_{t}^{⋆}\left(\theta_{0}^{⋆}\right)}{\partial \theta^{⋆\prime }}\right],\;\Omega =E\left(\frac{z_{t}z_{t}^{\prime }}{h_{t}^{2}}\right), \end{array}$$

$$\begin{array}{c} H^{⋆}=E\left[\frac{z_{t}}{h_{t}h_{t}^{⋆}\left(\theta_{0}^{⋆}\right)}\frac{\partial h_{t}^{⋆}\left(\theta_{0}^{⋆}\right)}{\partial \theta^{⋆\prime }}\right],\;\Gamma =E\left[\frac{z_{t}}{h_{t}^{2}}\sum_{j=1}^{p}\beta_{0j}\frac{\partial h_{t-j}\left(\theta_{0}\right)}{\partial \theta^{\prime }}\right], \end{array}$$

where $\theta_{0}^{\prime }=\theta_{0}^{⋆\prime }M$ and

(13) $$M=\mathrm{diag}\left\{\underset{q+1}{\underset{︸}{1/\mu^{2},\ldots ,1/\mu^{2}}},\underset{p}{\underset{︸}{1,\ldots ,1}}\right\}.$$

We next present the asymptotic behavior of the conditional quantile estimator ${\hat{\theta }}_{\tau n}^{⋆}$.

**Theorem** **1.** *Under assumptions (A1)–(A6), the estimator ${\hat{\theta }}_{\tau n}$ is asymptotically normal, i.e.,*

(14) $$\begin{array}{c} \sqrt{n}\left({\hat{\theta }}_{\tau n}^{⋆}-\theta_{\tau 0}\right)\overset{d}{⟶}N\left(0,\Sigma_{2}^{⋆}\right), \end{array}$$

*where $\theta_{\tau 0}=b_{\tau }\theta_{0}$, and*

(15) $$\Sigma_{2}^{⋆}=\Omega^{-1}\left[\frac{\tau -\tau^{2}}{f^{2}\left(b_{\tau }\right)}\Omega +\frac{\kappa_{1}^{⋆}b_{\tau }}{f\left(b_{\tau }\right)}\left(H^{⋆}J^{⋆-1}M^{-1}\Gamma^{\prime }+\Gamma M^{-1}J^{⋆-1}H^{⋆\prime }\right)+\kappa_{2}^{⋆}b_{\tau }^{2}\Gamma M^{-1}J^{⋆-1}M^{-1}\Gamma^{\prime }\right]\Omega^{-1}.$$

Compared with the unweighted procedure, the weighted approach adopted in Step M2 avoids the need for more restrictive moment conditions, which would otherwise shrink the admissible parameter space of the model. Moreover, the weighted method is known to yield more stable and reliable estimation results, as documented by Zheng et al. [15]. For these reasons, we employ the weighted procedure in Step M2 to construct the estimator ${\hat{\theta }}_{\tau n}^{⋆}$. Then, based on Theorem 1, the corresponding large-sample properties of the level-τ conditional quantile estimator for $y_{n+1}$ can then be established.

**Corollary** **1.** *Assuming that the conditions of Theorem 1 hold, the difference between the estimated and true conditional quantiles of $y_{n+1}$ admits the following stochastic expansion:*

$$\begin{array}{c} {\hat{Q}}_{\tau }^{⋆}\left(y_{n+1}|F_{n}\right)-Q_{\tau }\left(y_{n+1}|F_{n}\right)=u_{n+1}^{\prime }\left({\hat{\theta }}_{n}^{⋆}-\theta_{0}\right)+z_{n+1}^{\prime }\left({\hat{\theta }}_{\tau n}^{⋆}-\theta_{\tau 0}\right)+o_{p}\left(n^{-1/2}\right), \end{array}$$

*where*

(16) $$u_{n+1}=b_{\tau }\sum_{j=1}^{p}\beta_{0j}\frac{\partial h_{n+1-j}\left(\theta_{0}\right)}{\partial \theta }.$$

Furthermore, when $b_{\tau }\neq 0$, the estimator of the τ-th conditional quantile of $x_{n+1}$ can be expressed as:

$$\begin{array}{c} {\hat{Q}}_{\tau }^{⋆}\left(x_{n+1}|F_{n}\right)-Q_{\tau }\left(x_{n+1}|F_{n}\right)=\frac{u_{n+1}^{\prime }\left({\hat{\theta }}_{n}^{⋆}-\theta_{0}\right)+z_{n+1}^{\prime }\left({\hat{\theta }}_{\tau n}^{⋆}-\theta_{\tau 0}\right)}{2\sqrt{\left|b_{\tau }h_{n+1}\right|}}+o_{p}\left(n^{-1/2}\right). \end{array}$$

The above analysis establishes the large-sample properties of the conditional quantile estimators under the high-frequency augmented GARCH framework, thereby providing a theoretical foundation for subsequent statistical inference. Nevertheless, Theorem 1 indicates that the asymptotic covariance matrix $\Sigma_{2}^{⋆}$ depends on the unknown density function f(·) and the quantile-related constant $b_{\tau }$, both of which are difficult to estimate accurately in practice. To address this issue, and following Zheng et al. [15], we adopt a practical mixed bootstrap procedure to approximate the limiting distribution of the estimator ${\hat{\theta }}_{\tau n}^{⋆}$. The resulting bootstrap approximation facilitates the implementation of feasible inference and hypothesis testing in finite samples.

First, for computational convenience, we adopt the random-weight bootstrap procedure introduced by Jin et al. [24]. As shown in the proof of Theorem 1 in the Appendix A, the τ-th conditional quantile estimator ${\hat{\theta }}_{\tau n}^{*}$ depends explicitly on the preliminary QMELE estimator ${\hat{\theta }}_{n}^{⋆}$. In particular, it admits the stochastic expansion

$$\begin{array}{c} \sqrt{n}\left({\hat{\theta }}_{\tau n}^{⋆}-\theta_{\tau 0}\right)=\frac{\Omega^{-1}}{f\left(b_{\tau }\right)}T_{1n}-b_{\tau }\Omega^{-1}\Gamma \sqrt{n}\left({\hat{\theta }}_{n}^{⋆}-\theta_{0}\right)+o_{p}\left(1\right), \end{array}$$

where $T_{1n}=n^{-1/2}\sum_{t=1}^{n}\psi_{\tau }\left(\varepsilon_{t}-b_{\tau }\right)z_{t}/h_{t}$, with $\psi_{\tau }\left(x\right)=\tau -I\left(x<0\right)$. This representation indicates that both ${\hat{\theta }}_{n}^{⋆}$ and ${\hat{\theta }}_{\tau n}^{⋆}$ must be resampled within the bootstrap scheme. Accordingly, we apply the random-weighting bootstrap method to both estimators. The main steps of the procedure are summarized below.

- Step B1. In step M1, the random-weight bootstrap version of the QMELE procedure is applied to estimate the parameter vector $\theta^{⋆}$. Specifically, the bootstrap estimator is defined as
  $$\begin{array}{c} {\tilde{\theta }}_{n}^{*}=\underset{\theta \in \Theta^{⋆}}{\mathrm{argmin}}\sum_{t=1}^{n}\omega_{t}{\tilde{\ell }}_{t}^{⋆}\left(\theta \right), \end{array}$$
  in which the weights $\left\{\omega_{t}\right\}$ are non-negative i.i.d. random variables satisfying $E(\omega_{t})=1$ and $\mathrm{Var}(\omega_{t})=1$. The corresponding bootstrap estimate of the original parameter vector θ is then obtained as ${\hat{\theta }}_{n}^{*\prime }={\tilde{\theta }}_{n}^{\prime }\hat{M}$. Based on ${\hat{\theta }}_{n}^{*}$, the fitted conditional variances are subsequently computed and denoted by ${\tilde{h}}_{t}={\tilde{h}}_{t}\left({\hat{\theta }}_{n}^{*}\right)$.
- Step B2. Following Step M2, additional random weights are incorporated to construct the bootstrap version of the conditional quantile regression estimation. Specifically, the bootstrap estimator ${\hat{\theta }}_{\tau n}^{*}$ is obtained by solving
  $$\begin{array}{c} {\hat{\theta }}_{\tau n}^{*}=\underset{\theta_{\tau }}{\mathrm{argmin}}\sum_{t=1}^{n}\frac{\omega_{t}}{{\tilde{h}}_{t}^{⋆}}\rho_{\tau }\left(y_{t}-\theta_{\tau }^{\prime }{\tilde{z}}_{t}^{*}\right), \end{array}$$
  in which ${\tilde{z_{t}}}^{*}={\left(1,x_{t-1}^{2},\ldots ,x_{t-q}^{2},{\tilde{h}}_{t-1}^{*},\ldots ,{\tilde{h}}_{t-p}^{*}\right)}^{\prime }$. By construction, both ${\tilde{h}}_{t}^{⋆}$ and ${\tilde{z_{t}}}^{*}$ incorporate information from intraday high-frequency data through the volatility proxy.
- Step B3. Analogous to step M3, the τ-th conditional quantile of $\left\{x_{t}\right\}$ is estimated by
  $${\hat{Q}}_{\tau }^{*}\left(x_{t}|F_{t-1}\right)=T^{-1}\left({\hat{\theta }}_{\tau n}^{*\prime }{\tilde{z}}_{t}^{*}\right).$$

Moreover, following Zheng et al. [15], it can be shown that the bootstrap deviation of the preliminary QMELE estimator admits the expansion

$$\sqrt{n}\left({\tilde{\theta }}_{n}^{*}-{\tilde{\theta }}_{n}^{⋆}\right)=-\frac{J^{⋆-1}}{\sqrt{n}}\sum_{t=1}^{n}\left(\omega_{t}-1\right)\frac{1}{2}\left(1-\frac{H_{t}}{\sqrt{h_{t}^{⋆}\left(\theta_{0}^{⋆}\right)}}\right)\frac{1}{h_{t}^{⋆}\left(\theta_{0}^{⋆}\right)}\frac{\partial h_{t}^{⋆}\left(\theta_{0}^{⋆}\right)}{\partial \theta^{⋆}}+o_{p}\left(1\right).$$

In practice, the matrix $J^{⋆}$ can be consistently estimated by its sample analogue, i.e.,

$${\tilde{J}}^{⋆}=\frac{1}{n}\sum_{t=1}^{n}\frac{1}{{\tilde{h}}_{t}^{⋆2}\left({\tilde{\theta }}_{n}^{⋆}\right)}\left(\frac{\partial {\tilde{h}}_{t}^{⋆}\left({\tilde{\theta }}_{n}^{⋆}\right)}{\partial \theta^{⋆}}\right)\left(\frac{\partial {\tilde{h}}_{t}^{⋆}\left({\tilde{\theta }}_{n}^{⋆}\right)}{\partial \theta^{⋆\prime }}\right).$$

As a result, Step B1 can be replaced by the following computationally convenient alternative.

- Step $B1^{*}$. The estimator ${\hat{\theta }}_{n}^{*}$ is calculated consistent as
  (17) $$\begin{array}{c} {\hat{\theta }}_{n}^{*}={\hat{\theta }}_{n}^{⋆}-\hat{M}\left(\frac{{\tilde{J}}^{⋆-1}}{n}\sum_{t=1}^{n}\left(\omega_{t}-1\right)\frac{1}{2}\left(1-\frac{H_{t}}{\sqrt{h_{t}^{⋆}\left(\theta_{0}^{⋆}\right)}}\right)\frac{1}{h_{t}^{⋆}\left(\theta_{0}^{⋆}\right)}\frac{\partial h_{t}^{⋆}\left(\theta_{0}^{⋆}\right)}{\partial \theta^{⋆}}\right). \end{array}$$

Combining Steps B1^*^, B2 and B3 yields the proposed high-frequency augmented bootstrap procedure. Owing to the introduction of random weighting, an additional regularity condition is required to establish the asymptotic validity of the bootstrap approximation. Accordingly, we impose the following supplementary assumption.

**Assumption** **7.** *The i.i.d. non-negative random weights $\left\{\omega_{t}\right\}$ satisfy the moment condition $E{\left|\omega_{t}\right|}^{2+\kappa_{0}}<\infty$ for some constant $\kappa_{0}>0$.*

**Theorem** **2.** *Suppose that Assumptions 1–7 hold and $E{\left|\eta_{t}\right|}^{4+2\nu_{0}}<\infty$ for some $\nu_{0}>0$, then it follows that*

(18) $$\sqrt{n}\left({\hat{\theta }}_{\tau n}^{*}-{\hat{\theta }}_{\tau n}^{⋆}\right)\overset{d}{⟶}N\left(0,\Sigma_{2}^{⋆}\right),$$

*where the asymptotic variance–covariance matrix $\Sigma_{2}^{⋆}$ defined in (15).*

**Corollary** **2.** *Suppose that the conditions of Theorem 2 hold, then the bootstrap approximation error for the conditional quantile of $y_{n+1}$ admits the expansion*

$$\begin{array}{c} {\hat{Q}}_{\tau }^{*}\left(y_{n+1}|F_{n}\right)-{\hat{Q}}_{\tau }^{⋆}\left(y_{n+1}|F_{n}\right)=u_{n+1}^{\prime }\left({\hat{\theta }}_{n}^{*}-{\hat{\theta }}_{n}^{⋆}\right)+z_{n+1}^{\prime }\left({\hat{\theta }}_{\tau n}^{*}-{\hat{\theta }}_{\tau n}^{⋆}\right)+o_{p}\left(n^{-1/2}\right), \end{array}$$

*where $u_{n+1}$ is as defined in (16) as stated in Corollary 1.*

Based on Theorem 2, the asymptotic distribution of ${\hat{\theta }}_{\tau n}^{*}-{\hat{\theta }}_{\tau n}^{⋆}$ provides a valid approximation to that of ${\hat{\theta }}_{\tau n}^{⋆}-\theta_{\tau 0}$. This bootstrap-based approximation circumvents the need to directly estimate the unknown density function f(·), which is typically difficult to evaluate in practice. Moreover, the bootstrap replicates of ${\hat{Q}}_{\tau^{*}}\left(y_{n+1}|F_{n}\right)$ can be used to construct confidence intervals for $Q_{\tau }\left(y_{n+1}|F_{n}\right)$ The large-sample results further highlight the role of intraday high-frequency information in improving the bootstrap inference procedure. Since the transformation $T^{-1}\left(\cdot \right)$ is monotonic, it follows that

$$Q_{\tau }\left(x_{n+1}|F_{n}\right)=T^{-1}\left(Q_{\tau }\left(y_{n+1}|F_{n}\right)\right).$$

Accordingly, confidence intervals for $Q_{\tau }\left(x_{n+1}|F_{n}\right)$ can be constructed using the empirical quantiles of the bootstrap distribution

$${\hat{Q}}_{\tau }^{*}\left(x_{n+1}|F_{n}\right)=T^{-1}\left({\hat{Q}}_{\tau^{*}}\left(y_{n+1}|F_{n}\right)\right).$$

In Section 4, we use the asymptotic variance obtained by this bootstrap method to construct confidence intervals for the estimated parameters and calculate the coverage probability.

To summarize, the bootstrap approximation procedure can be implemented as follows.

- Step 1. Generate a sequence of non-negative i.i.d. random weights $\left\{\omega_{t}\right\}$ that satisfy $E\left(\omega_{t}\right)=\mathrm{Var}\left(\omega_{t}\right)=1$.
- Step 2. Obtain the estimators ${\hat{\theta }}_{n}^{⋆}$ and ${\hat{\theta }}_{\tau n}^{⋆}$ using Steps M1 and M2, respectively. Next, compute the bootstrap estimator ${\hat{\theta }}_{n}^{*}$ via step $B1^{*}$, and derive ${\hat{\theta }}_{\tau n}^{*}$ according to Step B2.
- Step 3. Compute
  $$A^{\left(1\right)}=\sqrt{n}\left({\hat{\theta }}_{\tau n}^{*}-{\hat{\theta }}_{\tau n}^{⋆}\right),\;\;Q^{\left(1\right)}=T^{-1}\left({\hat{\theta }}_{\tau n}^{*\prime }{\tilde{z}}_{n+1}^{*}\right).$$
  Repeat Steps 1 and 2 independently B−1 additional times to obtain the bootstrap samples
  $$\left\{A^{\left(1\right)},\ldots ,A^{\left(B\right)}\right\},\;\;\left\{Q^{\left(1\right)},\ldots ,Q^{\left(B\right)}\right\}.$$
  The limiting distribution of $\sqrt{n}\left({\hat{\theta }}_{\tau n}^{*}-\theta_{\tau 0}\right)$ is then approximated by the empirical distribution of ${\left\{A^{\left(i\right)}\right\}}_{i=1}^{B}$, while the empirical distribution of ${\left\{Q^{\left(i\right)}\right\}}_{i=1}^{B}$ is used to construct confidence intervals for $Q_{\tau }\left(x_{n+1}|F_{n}\right)$.

## 3. Diagnostic Checking

### 3.1. Diagnostic Checking for Conditional Quantile Model

This section conducts a diagnostic evaluation of the estimated conditional quantiles, following the framework of Zheng et al. [15], to illustrate how the proposed methodology can be implemented in practice. To this end, we define the weighted residuals as

$$\begin{array}{c} \varepsilon_{t,\tau }=\frac{y_{t}-Q_{\tau }\left(y_{t}|F_{t-1}\right)}{h_{t}}=\varepsilon_{t}-b_{\tau },\;t\in Z, \end{array}$$

where $y_{t}=T\left(x_{t}\right)$ and $\varepsilon_{t}=T\left(\eta_{t}\right)$. If the GARCH model (1) correctly specifies the conditional quantiles $Q_{\tau }\left(x_{t}|F_{t-1}\right)$ and $Q_{\tau }\left(y_{t}|F_{t-1}\right)$ at level τ, then the moment condition

$$E\left[\psi_{\tau }\left(\varepsilon_{t,\tau }\right)|F_{t-1}\right]=0$$

must be satisfied. Motivated by the absolute residual autocorrelation function proposed by Li and Li [25] and Li et al. [26], we define the quantile autocorrelation function (QACF) for the residual sequence $\left\{\varepsilon_{t,\tau }\right\}$ at lag *k* as:

$$\begin{array}{c} \rho_{k,\tau }={\mathrm{qcor}}_{\tau }(\varepsilon_{t,\tau },|\varepsilon_{t-k,\tau }\left|\right)=\frac{E[\psi_{\tau }\left(\varepsilon_{t,\tau }\right)|\varepsilon_{t-k,\tau }|]}{\sqrt{\left(\tau -\tau^{2}\right)\sigma_{a,\tau }^{2}}},\;k=1,2,\ldots , \end{array}$$

where $\sigma_{a,\tau }^{2}=\mathrm{Var}(|\varepsilon_{t,\tau }\left|\right)=E(|\varepsilon_{t,\tau }{\left|-\mu_{a,\tau }\right)}^{2}$ and $\mu_{a,\tau }=E(|\varepsilon_{t,\tau }\left|\right)$. Consequently, under the null hypothesis that model (1) correctly specifies the conditional quantile $Q_{\tau }\left(y_{t}|F_{t-1}\right)$, the QACF values $\rho_{k,\tau }$ should be equal to zero for all lags *k*. In the subsequent analysis, we construct a formal diagnostic test based on this QACF measure to assess the adequacy of the fitted conditional quantile model.

For a given τ∈(0,1), let ${\hat{\theta }}_{\tau n}$ denote the estimator of the conditional quantile parameter vector. Correspondingly, define the fitted volatility sequence and the associated quantile regression covariates as ${\tilde{h}}_{t}$ and $\tilde{z_{t}}$, respectively. Based on these quantities, the sample weighted residuals are constructed as

(19) $$\begin{array}{c} {\hat{\varepsilon }}_{t,\tau }=\frac{y_{t}-{\hat{\theta }}_{\tau n}^{\prime }\tilde{z_{t}}}{{\tilde{h}}_{t}},\;t=1,\ldots ,n. \end{array}$$

The sample QACF of the residuals at lag *k* is then given by

$$\begin{array}{c} r_{k,\tau }=\frac{1}{\sqrt{\left(\tau -\tau^{2}\right){\hat{\sigma }}_{a,\tau }^{2}}}\cdot \frac{1}{n}\sum_{t=k+1}^{n}\psi_{\tau }\left({\hat{\varepsilon }}_{t,\tau }\right)\left|{\hat{\varepsilon }}_{t-k,\tau }\right|, \end{array}$$

where

$${\hat{\sigma }}_{a,\tau }^{2}=\frac{1}{n}\sum_{t=1}^{n}{\left({\hat{\varepsilon }}_{t,\tau }|-{\hat{\mu }}_{a,\tau }\right)}^{2},\;\;{\hat{\mu }}_{a,\tau }=\frac{1}{n}\sum_{t=1}^{n}\left|{\hat{\varepsilon }}_{t,\tau }\right|.$$

Let $R={\left(r_{1,\tau },\ldots ,r_{K,\tau }\right)}^{\prime }$, where *K* is a fixed positive integer. Under the null hypothesis, the vector R converges to a mean-zero random vector, whereas under the alternative hypothesis its components are expected to exhibit systematic departures from zero. This contrast motivates the construction of a standardized test statistic based on *R*. As shown in Zheng et al. [15], $\sqrt{n}R$ converges in distribution to a multivariate normal random vector $N(0,\Sigma_{1})$, where $\Sigma_{1}$ is a positive-definite covariance matrix. Consequently, the quadratic form $Q\left(K\right)=nR^{\prime }{\hat{\Sigma }}_{1}^{-1}R$ converges in distribution to a chi-square random variable with *K* degrees of freedom as n→∞, where ${\hat{\Sigma }}_{1}$ denotes a consistent estimator of $\Sigma_{1}$. The same construction is adopted to develop a corresponding diagnostic test when high-frequency augmented data are employed.

### 3.2. Diagnostic Checking for High-Frequency Augmented Conditional Quantile Model

Building on the preceding theoretical results, the lag-*k* residual QACF based on high-frequency augmented data can be readily obtained. In analogy to (19), we have

$$\begin{array}{c} {\hat{\varepsilon }}_{t,\tau }^{⋆}=\frac{y_{t}-{\hat{\theta }}_{\tau n}^{⋆\prime }{{\tilde{z}}_{t}}^{⋆}}{{\tilde{h}}_{t}^{⋆}},\;t=1,\ldots ,n. \end{array}$$

Similarly, the sample weighted residual QACF is constructed under the high-frequency augmented framework, i.e.,

$$\begin{array}{c} r_{k,\tau }^{⋆}=\frac{1}{\sqrt{\left(\tau -\tau^{2}\right){\hat{\sigma }}_{a,\tau }^{⋆2}}}\cdot \frac{1}{n}\sum_{t=k+1}^{n}\psi_{\tau }\left({\hat{\varepsilon }}_{t,\tau }^{⋆}\right)\left|{\hat{\varepsilon }}_{t-k,\tau }^{⋆}\right|, \end{array}$$

in which

$${\hat{\sigma }}_{a,\tau }^{⋆2}=\frac{1}{n}\sum_{t=1}^{n}\left(\right|{\hat{\varepsilon }}_{t,\tau }^{⋆}|-{\hat{\mu }}_{a,\tau }^{⋆}{)}^{2},\;\;{\hat{\mu }}_{a,\tau }^{⋆}=\frac{1}{n}\sum_{t=1}^{n}\left|{\hat{\varepsilon }}_{t,\tau }^{⋆}\right|.$$

We begin by analyzing the asymptotic behavior of $R^{⋆}={\left(r_{1,\tau }^{⋆},\ldots ,r_{K,\tau }^{⋆}\right)}^{\prime }$, as this characterization provides the theoretical foundation for the hypothesis tests developed later. For this purpose, we define

$$\epsilon_{t}=\left(\right|\varepsilon_{t,\tau }\left|,\right|\varepsilon_{t-1,\tau }\left|,\ldots ,\right|\varepsilon_{t-K+1,\tau }{\left|\right)}^{\prime },\;\Xi =E\left(\epsilon_{t}\epsilon_{t}^{\prime }\right),$$

and then denote

$$\begin{array}{c} D_{1}=E\left(\frac{\epsilon_{t-1}z_{t}^{\prime }}{h_{t}}\right),\;D_{2}=E\left[\frac{\epsilon_{t-1}}{h_{t}}\sum_{j=1}^{p}\beta_{0j}\frac{\partial h_{t-j}\left(\theta_{0}\right)}{\partial \theta^{\prime }}\right],\;D_{3}=E\left[\frac{\epsilon_{t-1}}{h_{t}}\frac{\partial h_{t}\left(\theta_{0}\right)}{\partial \theta^{\prime }}\right]. \end{array}$$

To streamline the exposition, let $P=D_{2}-D_{1}\Omega^{-1}\Gamma$, $Q^{⋆}=D_{3}-D_{1}\Omega^{-1}H^{⋆}$ and $\Lambda =D_{1}\Omega^{-1}D_{1}^{\prime }$. We then obtain the following result.

**Theorem** **3.** *Suppose that $E{\left|\eta_{t}\right|}^{4+2\nu_{0}}<\infty$ for some $\nu_{0}>0$ and Assumptions 1–6 are satisfied, then it follows that*

$$\begin{array}{c} \sqrt{n}R^{⋆}\overset{d}{⟶}N\left(0,\Sigma_{3}^{⋆}\right), \end{array}$$

*in which*

(20) $$\Sigma_{3}^{⋆}=\frac{\Xi -\Lambda }{\sigma_{a,\tau }^{2}}+\frac{\kappa_{1}^{⋆}b_{\tau }f\left(b_{\tau }\right)}{\left(\tau -\tau^{2}\right)\sigma_{a,\tau }^{2}}\left(Q^{⋆}J^{⋆-1}P^{\prime }+PJ^{⋆-1}Q^{⋆\prime }\right)+\frac{\kappa_{2}^{⋆}b_{\tau }^{2}f^{2}\left(b_{\tau }\right)}{\left(\tau -\tau^{2}\right)\sigma_{a,\tau }^{2}}PJ^{⋆-1}P^{\prime }.$$

Based on Theorem 3, given a consistent estimator ${\hat{\Sigma }}_{3}^{⋆}$ for the covariance matrix $\Sigma_{3}^{⋆}$, the test statistic can be defined as:

$$\begin{array}{c} Q^{⋆}\left(K\right)=nR^{⋆\prime }{\hat{\Sigma }}_{3}^{⋆-1}R^{⋆}. \end{array}$$

Under the null hypothesis, $Q^{⋆}\left(K\right)$ converges in distribution to a chi-square random variable with *K* degrees of freedom as n→+∞. In practice, however, direct estimation of $\Sigma_{3}^{*}$ is challenging due to its dependence on the unknown density function f(·). To circumvent this difficulty, we adopt the bootstrap procedure developed in Section 2.2 to obtain a feasible approximation of $\Sigma_{3}^{⋆}$.

Let ${\hat{\varepsilon }}_{t,\tau }^{*}=\left(y_{t}-{\hat{\theta }}_{\tau n}^{*\prime }{{\tilde{z}}_{t}}^{*}\right)/{\tilde{h}}_{t}^{*}$, subsequently, the lag-*k* QACF for the randomly weighted residuals is calculated as

(21) $$\begin{array}{c} r_{k,\tau }^{*}=\frac{1}{\sqrt{\tau -\tau^{2}){\hat{\sigma }}_{a,\tau }^{*2}}}\cdot \frac{1}{n}\sum_{t=k+1}^{n}\omega_{t}\psi_{\tau }\left({\hat{\varepsilon }}_{t,\tau }^{*}\right)\left|{\hat{\varepsilon }}_{t-k,\tau }^{*}\right|. \end{array}$$

Denote $R^{*}={\left(r_{1,\tau }^{*},\ldots ,r_{K,\tau }^{*}\right)}^{\prime }$. The following theorem establishes the theoretical basis for the bootstrap test.

**Theorem** **4.** *Suppose that all assumptions stated in Theorem 2 hold, then it is true that*

$$\begin{array}{c} \sqrt{n}\left(R^{*}-R^{⋆}\right)\overset{d}{⟶}N\left(0,\Sigma_{3}^{*}\right),\;n\to \infty , \end{array}$$

*in which $\Sigma_{3}^{⋆}$ is defined by (20).*

Using Step 3 of the bootstrap procedure described in Section 2.2 the vector $R^{*}$ is computed according to Equation (21), and then the quantity $T^{\left(1\right)}=\sqrt{n}\left(R^{*}-R^{⋆}\right)$ is obtained. This procedure is repeated independently B−1 additional times, yielding the bootstrap sample $\left\{T^{\left(1\right)},\ldots ,T^{\left(B\right)}\right\}$. The asymptotic covariance matrix $\Sigma_{3}^{⋆}$ is then estimated by the sample covariance matrix of ${\left\{T^{\left(i\right)}\right\}}_{i=1}^{B}$, based on which the bootstrap test statistic $Q^{⋆}\left(K\right)$ is computed consequently. Finally, the null hypothesis, stating that the residual autocorrelation $r_{k,\tau }$ are jointly insignificant for 1⩽k⩽K, is rejected if $Q^{⋆}\left(K\right)$ exceeds the 95% critical value of the $\chi_{K}^{2}$ distribution.

## 4. Simulation

In this section, we conduct Monte Carlo simulations to assess the finite-sample performance of the proposed methodology. We first investigate the estimation accuracy of the parameter vector $\theta_{\tau }$, with particular emphasis on the effectiveness of the mixed bootstrap procedure in approximating its asymptotic distribution. We then evaluate the accuracy of conditional quantile forecasts and perform diagnostic checks for the underlying GARCH specification. To highlight the benefits of incorporating intraday information, the results based on high-frequency augmented data are compared with those obtained using only daily observations. All simulation experiments are carried out under the GARCH(1,1) framework.

### 4.1. Estimation Results Using High-Frequency Augmented Data

Following the simulation design of Visser [2], we generate intraday high-frequency observations through an Ornstein–Uhlenbeck-type stochastic volatility mechanism for the process $Z_{t}\left(u\right)$. Specifically, consider

(22) $$\begin{array}{cc} d\Gamma_{t}\left(u\right) & =-\delta \left(\Gamma_{t}\left(u\right)-\mu_{\Gamma }\right)du+\sigma_{\Gamma }dB_{t}^{\left(2\right)}\left(u\right), \end{array}$$

(23) $$\begin{array}{cc} d\Psi_{t}\left(u\right) & =\exp\left(\Gamma_{t}\left(u\right)\right)dB_{t}^{\left(1\right)}\left(u\right),u\in \left[0,1\right], \end{array}$$

(24) $$\begin{array}{cc} m & =E[|\Psi_{t}\left(1\right)\left|\right],\;\;\;\;\;\;Z_{t}\left(u\right)=\frac{\Psi_{t}\left(u\right)}{m} \end{array}$$

in which the processes $B_{t}^{\left(1\right)}\left(\cdot \right)$ and $B_{t}^{\left(2\right)}\left(\cdot \right)$ are independent Wiener processes; $\Gamma_{t}\left(u\right)∼N\left(\mu_{\Gamma },\sigma_{\Gamma }^{2}\right)$; $\Psi_{t}\left(0\right)=0$, $\Gamma_{t}\left(0\right)$ is drawn from the invariant distribution of $\Gamma_{t}\left(u\right)$. In practice, the normalization constant *m* in Equation (24) is estimated by $\hat{m}=n^{-1}\sum_{t=1}^{n}\left|\Psi_{t}\left(1\right)\right|$, which guarantees the standardization condition $E|Z_{t}\left(1\right)|=1$. To approximate one-minute intraday observations in an actual trading environment, assuming a four-hour trading session as in the Chinese stock market, the interval [0,1] is divided into 240 equally spaced sub-intervals. The parameters are fixed at δ=1/2, $\sigma_{\Gamma }=1/4$ and $\mu_{\Gamma }=-1/16$, yielding a discrete-time standardized process $Z_{t}\left(u\right)$. Based on this construction, intraday returns $X_{t}\left(u\right)$ are generated according to the high-frequency augmented GARCH model (6) with p=q=1. The observation at u=1 corresponds to the daily returns $\left\{x_{t}\right\}$. Throughout the simulation study, the quantile level is set to τ=0.1.

For parameter estimation, the volatility proxy H(·) is specified as the realized volatility (RV) constructed from intraday returns at different sampling frequencies, namely 5-, 15- and 30-min intervals, denoted by $\mathrm{RV}5_{t}$, $\mathrm{RV}15_{t}$ and $\mathrm{RV}30_{t}$, respectively. As an illustration, consider the simulated intraday observations ${\left\{X_{t}\left(u_{i}\right)\right\}}_{i=1}^{240}$. The 5-min realized volatility is computed as

$$\begin{array}{c} {RV5}_{t}=H\left(X_{t}\left(u\right)\right)={\left(\sum_{i=1}^{48}{\left[X_{t}\left(u_{5i}\right)-X_{t}\left(u_{5(i-1)}\right)\right]}^{2}\right)}^{1/2}, \end{array}$$

where $Y_{t}\left(u_{0}\right)=Y_{t}\left(0\right)=0$. The definitions of $\mathrm{RV}15_{t}$ and $\mathrm{RV}30_{t}$ follow analogously.

To estimate the auxiliary parameter μ required in the subsequent procedure, we follow the approach of Zhang et al. [22]. Specifically, we consider the degenerate case of the volatility proxy in model (7), where the absolute value of the daily return is used as a special volatility proxy, that is,

$$H_{t}=H\left(X_{t}\left(u\right)\right)=|X_{t}\left(1\right)|=|x_{t}|,$$

which corresponds to using daily returns only. In this case, we have

$$z_{H,t}=H\left(Z_{t}\left(u\right)\right)=|Z_{t}\left(1\right)|=|\eta_{t}|,\;\;\mu =E\left(z_{H,t}\right)=E(|Z_{t}\left(1\right)\left|\right)=1.$$

Consequently, the estimator obtained from Equation (11) coincides with ${\tilde{\theta }}_{n}$ in (5), namely the QMLE estimator based solely on daily data. Recalling the relationship $\sqrt{h_{t}^{⋆}}=\sqrt{h_{t}}\mu$, it follows that $\mu^{2}=h_{t}^{⋆}/h_{t}$. This suggests the estimator

$${\hat{\mu }}^{2}=\frac{1}{n}\sum_{t=1}^{n}\frac{{\tilde{h}}_{t}^{⋆}}{{\tilde{h}}_{t}},$$

where ${\tilde{h}}_{t}$ and ${\tilde{h}}_{t}^{⋆}$ are obtained from Steps E1 and M1, respectively. Moreover, it can be shown that $\hat{\mu }$ is a consistent estimator of μ similar to the result in Zhang et al. [22]. It should be noted that a deeper theoretical justification of the practical implications of estimating μ remains an interesting topic for future research.

To examine the finite-sample performance of the estimator ${\hat{\theta }}_{\tau n}^{⋆}$ and to assess the accuracy of the mixed bootstrap procedure in approximating its sampling distribution, we conduct two Monte Carlo simulation studies. Estimator performance is evaluated using three criteria: the bias of ${\hat{\theta }}_{n}^{⋆}$, defined as the difference between the estimate and the true parameter value, the empirical standard deviation (ESD) and the asymptotic standard deviation (ASD). We also calculate the coverage probability of each parameter confidence interval based on the asymptotic results obtained in Section 3. The data are generated from a GARCH(1,1) model with true parameter vector $\theta ={\left(\alpha_{0},\alpha_{1},\beta_{1}\right)}^{\prime }={\left(0.4,0.4,0.4\right)}^{\prime }$ and $\theta ={\left(\alpha_{0},\alpha_{1},\beta_{1}\right)}^{\prime }={\left(0.4,0.3,0.4\right)}^{\prime }$. We consider sample sizes n=500, 1000 and 2000. For each configuration, all reported results are based on averages over 1000 Monte Carlo replications.

Following Zheng et al. [15], the random weights $\omega_{t}$ used in the mixed bootstrap procedure are generated from three alternative distributions. The first is the standard exponential distribution, denoted by $W_{1}$; The second is a Rademacher-type distribution $W_{2}$, defined by $W_{2}$, with $P\left(W_{2}=0\right)=P\left(W_{2}=2\right)=0.5$. The third is Mammen’s two-point distribution $W_{3}$, specified as

$$P\left(W_{3}=\frac{-\sqrt{5}+3}{2}\right)=\frac{\sqrt{5}+1}{2\sqrt{5}},\;\;P\left(W_{3}=\frac{\sqrt{5}+3}{2}\right)=1-\frac{\left(\sqrt{5}+1\right)}{2\sqrt{5}}.$$

Table 1 and Table 2 report the bias of the estimator ${\hat{\theta }}_{n}^{⋆}$, together with the empirical standard deviation (ESD) and asymptotic standard deviation (ASD) of ${\hat{\theta }}_{\tau n}^{⋆}$, for both the high-frequency augmented and low-frequency models. All the ASD values are obtained using the proposed mixed bootstrap procedure. The results indicate that the estimation bias remains uniformly small across all configurations. As the sample size *n* increases, both ESD and ASD decrease substantially and converge toward each other, suggesting that the bootstrap approximation accurately captures the sampling variability. Moreover, the choice of random weighting scheme has little influence on the large-sample performance of the estimators. In contrast, for smaller sample sizes, different weight distributions may lead to noticeable discrepancies in ASD values, highlighting the greater sensitivity of finite-sample behavior to the choice of bootstrap weights. The coverage probability (CP) gradually approaches the nominal level of 95% as the sample size increases. It is also observed that the CP under the high-frequency setting is generally closer to 95% than that under the daily-frequency setting, particularly when the sample size is small.

It is also worth noting that the use of high-frequency augmented data improves the precision of GARCH parameter estimation, as evidenced by the smaller ESD and ASD values of ${\hat{\theta }}_{\tau n}^{⋆}$. Nevertheless, the mixed-frequency approach does not always yield a smaller bias than the low-frequency method when estimating θ. This discrepancy arises because Zheng et al. [15] assume normally or Student-t distributed innovations, whereas in our setting the standardized process $Z_{t}\left(u\right)$ is generated through Equations (22) and (23), whose distributional properties are analytically less tractable. Consequently, the true value of $b_{\tau }$ in the relation $\theta_{\tau }=b\tau {\left(\alpha_{0},\alpha_{1},\beta_{1}\right)}^{\prime }$ is not directly available. To address this issue, we adopt the following approximation strategy. First, we compute ${\hat{\theta }}_{\tau n}$, and then rescale each of its components by the corresponding true parameter values $\alpha_{00}$, $\alpha_{01}$ and $\beta_{01}$. The average of these three ratios is taken as an estimator of $b_{\tau }$. Repeating this procedure across 1000 Monte Carlo replications and averaging the resulting estimates yields an approximation of the true $b_{\tau }$. Using this value, the elements of ${\hat{\theta }}_{\tau n}$ are adjusted to obtain the final parameter estimates. As reported in Table 1 and Table 2, even without direct knowledge of the true $b_{\tau }$, the bias of the mixed-frequency estimator remains comparable to that obtained under the low-frequency framework.

### 4.2. Forecast Results Using High-Frequency Augmented Data

While parameter estimation in GARCH models is important, forecasting conditional quantiles is often of greater practical interest. To assess the predictive performance of the proposed approach, we conduct both in-sample and out-of-sample evaluations. For the in-sample analysis, we examine the conditional quantiles $Q_{\tau }\left(x_{t}|F_{t-1}\right)$ for 1≤t≤n, whereas the out-of-sample performance is assessed using $Q\tau (x_{n+1}|F_{n})$. We compare the forecast bias and mean squared error (MSE) of conditional quantile estimates obtained from the high-frequency augmented approach with those derived from the conventional low-frequency method. The simulation data are generated under two distinct parameter settings, described as follows:

Scenario 1: $\theta_{0}={\left(\alpha_{0},\alpha_{1},\beta_{1}\right)}^{\prime }={\left(0.1,0.25,0.6\right)}^{\prime }$;

Scenario 2: $\theta_{0}={\left(\alpha_{0},\alpha_{1},\beta_{1}\right)}^{\prime }={\left(0.1,0.2,0.7\right)}^{\prime }$.

Sample sizes are set to n=100,200 and 500, with 1000 Monte Carlo replications conducted for each case. For each configuration, the accuracy of conditional quantile forecasts, which is measured by bias and mean squared error (MSE), is evaluated by averaging the corresponding statistics across all replications. The results are reported in Table 3 and Table 4. Since the distribution of $Z_{t}\left(u\right)$ in (6) does not admit a closed-form expression, the true conditional quantiles of $\left\{x_{t}\right\}$ are approximated using a large simulated sample of size 10,000. We further verify that increasing the sample size to 20,000 or 50,000 yields nearly identical quantile estimates, confirming the adequacy of this approximation.

Three main findings emerge from these tables. First, for both the high-frequency augmented and low-frequency approaches, smaller in-sample bias is generally associated with improved out-of-sample performance. Second, incorporating intraday high-frequency information into the GARCH framework substantially enhances the accuracy of conditional quantile forecasts: the high-frequency augmented approach typically yields smaller bias than the conventional low-frequency method. Among the various sampling intervals considered, the 15-min realized volatility consistently produces the smallest bias. Third, the patterns observed for MSE closely mirror those for bias. In particular, the MSE values obtained from the high-frequency augmented method are markedly lower than those from the low-frequency approach, with the 15-min realized volatility again delivering the best overall performance. Moreover, MSE decreases steadily with increasing sample size for both modeling strategies.

Overall, the high-frequency augmented approach delivers more accurate conditional quantile forecasts than the low-frequency model, with the advantage being especially pronounced in smaller samples. As shown in Table 3 and Table 4, forecast performance improves substantially as the sample size increases from n=100 to n=200. With n=500, the high-frequency augmented method yields stable and reliable estimates, highlighting its potential usefulness in empirical applications.

### 4.3. Diagnostic Checking Using High-Frequency Augmented Data

In this subsection, we examine the empirical size and power properties of the test statistic $Q^{⋆}\left(K\right)$, which is constructed using the proposed bootstrap procedure. The performance of the test based on high-frequency augmented data is compared with that obtained using only daily observations. The data are generated according to the following process:

$$\left\{\begin{array}{c} X_{t}\left(u\right)=\sqrt{h_{t}}Z_{t}\left(u\right),\;0\leq u\leq 1,\\ h_{t}=0.4+0.2x_{t-1}^{2}+dx_{t-4}^{2}+0.2h_{t-1}. \end{array}\right.$$

where $Z_{t}\left(u\right)$ is generated according to Equations (22) and (23), with deviation parameters set to d=0, 0.3 and 0.6. Since the analysis focuses on conditional quantile estimation for the GARCH(1,1) model, the case d=0 corresponds to evaluating the empirical size of the test, whereas nonzero values of *d* are used to assess its power. All other simulation settings are identical to those employed in the previous experiments. The rejection rates of the null hypothesis are reported based on 1000 Monte Carlo replications at the 5% significance level.

Table 5 reports the rejection rates of the test statistic $Q^{⋆}\left(K\right)$ with the maximum lag set to K=6. Overall, both the size and power properties of the test are satisfactory. As the sample size *n* increases, the empirical rejection rates under the null hypothesis converge toward the nominal 5% level. Even for relatively small sample sizes, the test based on high-frequency augmented data consistently outperforms its counterpart based solely on daily observations. Test power increases with either larger sample sizes or higher deviation values *d*. Across all configurations, the high-frequency augmented test exhibits uniformly stronger power than the daily-data-based test. Higher power reflects a greater ability to correctly reject the null hypothesis when it is violated, that is, when the vector *R* deviates significantly from zero. This advantage becomes particularly pronounced at n=2000, where the gap in power between the two approaches is substantial. Moreover, the choice of random weight distributions in the mixed bootstrap procedure has only a negligible effect on the rejection rates, a pattern that is consistent with the findings from the parameter estimation results.

## 5. Empirical Application

This section applies the proposed model and methodology to real financial data. We analyze the logarithmic return series of three major Chinese stock market indices: the CSI 300, SSE 50 and CSI 500 indices, observed over the periods 8 April 2005–6 June 2019, 2 January 2004–6 June 2019 and 15 January 2007–6 June 2019, respectively. These empirical examples are used to illustrate the practical performance of the high-frequency augmented framework. Taking the CSI 300 Index as a representative example, the dataset consists of 1-min closing prices over the period from 8 April 2005 to 6 June 2019, covering a total of 3441 trading days, with 241 intraday observations per day. Let $\left\{P_{t}\left(u\right),t\in \left[0,3441\right],u\in \left[0,1\right]\right\}$ denote the intraday price process. The intraday high-frequency logarithmic return at time *u* on day *t* is defined as

$$\begin{array}{c} Y_{t}\left(u\right)=\left(\log P_{t}\left(u\right)-\log P_{t-1}\left(1\right)\right)\times 100. \end{array}$$

**Figure 1:**
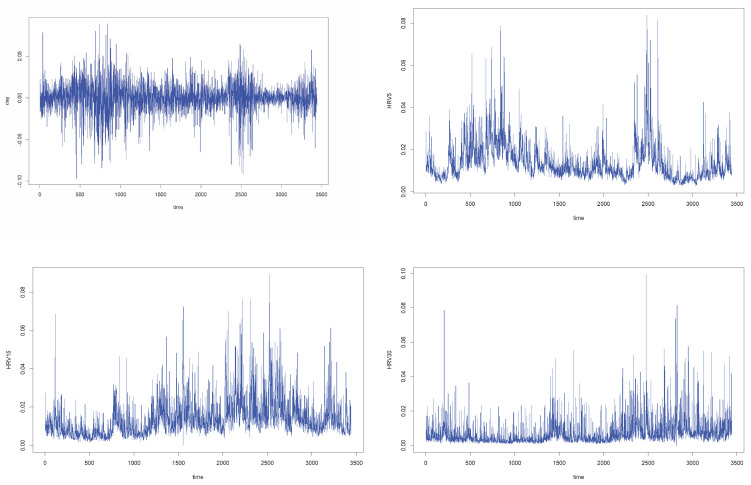
Time series of the CSI 300 Index and selected volatility proxies.

The corresponding low-frequency returns, namely the daily logarithmic returns $Y_{t}\left(1\right)$, constitute the series $x_{t}$ used in the low-frequency GARCH model estimation. For volatility proxy construction, we employ realized volatility measures computed at 5-, 15- and 30-min sampling intervals, denoted by RV5, RV15 and RV30, respectively. For comparison, we also consider the absolute daily return $H_{t}=\left|x_{t}\right|$. which does not incorporate intraday high-frequency information. Figure 1 displays the daily log-returns $x_{t}$ together with the various volatility proxies for the CSI 300 Index. To assess the forecasting performance of the proposed method, we focus on two quantile levels: τ=0.05 and τ=0.01, corresponding to one-day 5% and 1% Value-at-Risk (VaR), respectively. In implementing the mixed bootstrap procedure, the random weights are generated from the standard exponential distribution. and we consider GARCH(1,1) model for $\left\{x_{t}\right\}$ and estimate the conditional quantiles using the proposed estimation procedure.

To further evaluate the forecasting performance of the proposed approach, we estimate the 5% conditional quantile (5% VaR) of daily log-returns. Using the CSI 300 Index as an illustrative example, we first fit a GARCH(1,1) model over an initial two-year estimation window, spanning from 8 April 2005 to 6 April 2007. Based on the fitted model, we compute the one-step-ahead conditional quantile $Q_{\tau }\left(x_{n+1}|F_{n}\right)$ for the next trading day. The estimation window is then expanded by one observation, and the forecasting procedure is repeated in a rolling fashion until the end of the sample period. After completing the rolling forecasts, predictive accuracy is assessed using the empirical coverage error, defined as the difference between the realized coverage rate (i.e., the proportion of observed returns falling below the predicted quantile) and the nominal quantile level τ.

Table 6 reports the empirical coverage errors for the low-frequency model and for the high-frequency augmented specifications based on 5-, 15- and 30-min realized volatility measures. Over the full sample period, the high-frequency augmented approach consistently outperforms the low-frequency alternative, as evidenced by smaller absolute coverage errors at both the 1% and 5% VaR levels across all three indices. For example, in the case of the CSI 300 Index, the low-frequency model yields a coverage error of 0.62% for the 1% VaR, whereas the high-frequency augmented models produce errors of 0.21%, 0.20% and 0.25%, respectively. These values are substantially closer to zero, indicating more accurate calibration to the nominal quantile level. To further facilitate comparison between two estimation approaches, Table 6 also reports the empirical coverage errors obtained using the QMLE method. The results show that, except for the CSI 300 Index where the two methods perform similarly, the QMELE-based estimates generally produce smaller empirical coverage errors than those based on QMLE in the other two stock markets. Overall, the empirical results highlight the practical relevance and improved forecasting accuracy of our proposed methodology.

Finally, Table 7 illustrates the parameter estimates together with their corresponding standard errors for the high-frequency augmented model based on the RV5 volatility proxy (the standard errors are reported in parentheses to the right of the parameter estimates). Among the realized volatility proxies considered in this study, RV5 is selected because it provides the best performance in terms of empirical coverage errors for quantile prediction.

## 6. Conclusions

This paper develops a conditional quantile estimation method for standard GARCH models by integrating quasi-maximum exponential likelihood estimation (QMELE) with high-frequency augmented data. The proposed approach improves estimation accuracy relative to conventional low-frequency methods. We construct estimators for both model parameters and conditional quantiles and establish their asymptotic properties. Extensive simulation studies confirm the effectiveness of the method in finite samples, while empirical applications to three major stock indices demonstrate its strong practical performance. Several extensions merit future investigation. The proposed method can be adapted to other heteroskedastic models, including nonparametric, threshold and multivariate GARCH specifications. In addition, incorporating composite quantile regression within the high-frequency augmented setting may further enhance estimation efficiency and robustness. On the other hand, it is also worth noting that high-frequency financial data may be affected by microstructure noise arising from market frictions such as bid–ask spreads and price discreteness. Such noise may influence the accuracy of volatility proxies constructed from high-frequency observations and, consequently, affect statistical inference in augmented GARCH-type models. A systematic investigation of the sensitivity of the proposed framework to microstructure noise s an interesting topic and will be considered in our future research.

## Figures and Tables

**Table 1:** Bias, ESD and ASD at τ=0.1 with $\alpha_{0}=0.4,\;\alpha_{1}=0.3,\;\beta_{1}=0.4$, where ${\mathrm{ASD}}_{i}$ corresponds to the random weight $W_{i}$, i=1,2,3.

Parameter	Volatility Proxy	n=500						n=1000						n=2000					
		Bias	ESD	ASD_1_	ASD_2_	ASD_3_	CP	Bias	ESD	ASD_1_	ASD_2_	ASD_3_	CP	Bias	ESD	ASD_1_	ASD_2_	ASD_3_	CP
$\alpha_{0}$	day	−0.0830	0.5170	0.4957	0.5068	0.5058	0.873	−0.0509	0.4336	0.4462	0.4492	0.4509	0.906	−0.0052	0.3036	0.3088	0.3053	0.3050	0.919
	RV5	−0.0285	0.4092	0.3949	0.4267	0.4152	0.916	0.0150	0.3168	0.3085	0.3036	0.3071	0.925	0.0033	0.2176	0.2144	0.2122	0.2125	0.940
	RV15	−0.0301	0.3901	0.3923	0.4073	0.4221	0.912	0.0178	0.3423	0.3305	0.3155	0.3212	0.924	0.0039	0.2327	0.2228	0.2202	0.2213	0.941
	RV30	−0.0312	0.4047	0.4115	0.4303	0.4231	0.920	0.0192	0.3516	0.3424	0.3719	0.3611	0.931	0.0036	0.2308	0.2330	0.2300	0.2308	0.941
$\alpha_{1}$	day	−0.0751	0.2838	0.2787	0.2654	0.2748	0.881	−0.0549	0.2727	0.2429	0.2535	0.2533	0.912	−0.0069	0.2271	0.2280	0.2223	0.2281	0.927
	RV5	−0.0252	0.2121	0.2006	0.2524	0.2589	0.909	−0.0166	0.1776	0.1790	0.1801	0.1810	0.918	−0.0039	0.1284	0.1271	0.1266	0.1265	0.942
	RV15	−0.0285	0.2108	0.1998	0.2114	0.2280	0.911	−0.0175	0.1724	0.1783	0.1795	0.1804	0.920	−0.0042	0.1297	0.1267	0.1261	0.1260	0.938
	RV30	−0.0307	0.2004	0.1989	0.2211	0.2271	0.906	−0.0182	0.1786	0.1776	0.1789	0.1797	0.921	−0.0043	0.1222	0.1263	0.1258	0.1257	0.941
$\beta_{1}$	day	−0.1058	0.4836	0.4666	0.4616	0.4620	0.902	0.0651	0.4406	0.4266	0.4284	0.4299	0.924	0.0122	0.2846	0.2951	0.2949	0.2946	0.932
	RV5	−0.0611	0.3992	0.3899	0.4175	0.4222	0.924	−0.0207	0.3238	0.3272	0.3145	0.3220	0.935	−0.0055	0.2284	0.2276	0.2249	0.2254	0.942
	RV15	−0.0663	0.4068	0.3946	0.4112	0.4272	0.927	−0.0232	0.3433	0.3503	0.3254	0.3357	0.936	−0.0057	0.2386	0.2356	0.2324	0.2336	0.945
	RV30	−0.0653	0.3985	0.3996	0.4203	0.4371	0.921	−0.0218	0.3516	0.3607	0.3754	0.3513	0.932	−0.0069	0.2402	0.2446	0.2408	0.2418	0.944

**Table 2:** Bias, ESD and ASD at τ=0.1 with $\alpha_{0}=0.4,\;\alpha_{1}=0.4,\;\beta_{1}=0.4$, where ${\mathrm{ASD}}_{i}$ corresponds to the random weight $W_{i}$, i=1,2,3.

Parameter	Volatility Proxy	n=500						n=1000						n=2000					
		Bias	ESD	ASD_1_	ASD_2_	ASD_3_	CP	Bias	ESD	ASD_1_	ASD_2_	ASD_3_	CP	Bias	ESD	ASD_1_	ASD_2_	ASD_3_	CP
$\alpha_{0}$	day	0.0630	0.5592	0.5314	0.5321	0.5344	0.895	−0.0185	0.4129	0.4065	0.4081	0.4045	0.916	−0.0073	0.2778	0.2743	0.2746	0.2745	0.93
	RV5	−0.0217	0.4676	0.4526	0.4253	0.4325	0.92	−0.0095	0.3092	0.2949	0.2937	0.2989	0.931	−0.0034	0.2014	0.2085	0.2069	0.2071	0.942
	RV15	−0.0229	0.4903	0.4799	0.4374	0.4426	0.918	−0.0101	0.3071	0.3023	0.3010	0.3067	0.928	−0.0035	0.2298	0.2135	0.2125	0.2126	0.94
	RV30	0.0204	0.4936	0.4829	0.4494	0.4737	0.92	−0.0112	0.3187	0.3115	0.3097	0.3167	0.93	−0.0030	0.2112	0.2199	0.2188	0.2193	0.938
$\alpha_{1}$	day	−0.0627	0.4519	0.4053	0.4127	0.4054	0.906	−0.0166	0.3369	0.3237	0.3156	0.3250	0.918	−0.0079	0.2190	0.2084	0.2026	0.2025	0.929
	RV5	−0.0206	0.3012	0.2894	0.2855	0.2911	0.919	−0.0092	0.2092	0.2006	0.2014	0.2011	0.927	−0.0036	0.1516	0.1422	0.1405	0.1421	0.941
	RV15	−0.0194	0.3090	0.2883	0.2842	0.2894	0.916	−0.0097	0.2008	0.1998	0.2004	0.2002	0.925	−0.0037	0.1447	0.1415	0.1399	0.1415	0.941
	RV30	−0.0237	0.3049	0.2869	0.2827	0.2883	0.92	−0.0107	0.2004	0.1989	0.1994	0.1994	0.926	−0.0039	0.1465	0.1410	0.1392	0.1408	0.94
$\beta_{1}$	day	−0.0558	0.5687	0.5105	0.5213	0.5187	0.911	0.0189	0.4006	0.3850	0.3847	0.3809	0.92	0.0091	0.2954	0.2882	0.2936	0.2935	0.936
	RV5	−0.0162	0.4508	0.4299	0.4123	0.4226	0.926	−0.0095	0.2992	0.2899	0.2854	0.2916	0.937	−0.0052	0.2086	0.2038	0.2007	0.2041	0.944
	RV15	−0.0173	0.4701	0.4474	0.4206	0.4286	0.924	−0.0096	0.3008	0.2946	0.2907	0.2970	0.935	−0.0055	0.2090	0.2069	0.2046	0.2081	0.94
	RV30	−0.0177	0.4634	0.4489	0.4266	0.4608	0.927	−0.0095	0.3085	0.2996	0.2951	0.3031	0.932	−0.0053	0.2130	0.2102	0.2082	0.2119	0.942

**Table 3:** Bias and MSE of the conditional quantile forecasts under the parameter setting $\tau =0.05,\alpha_{0}=0.1,\;\alpha_{1}=0.25$ and $\beta_{1}=0.6$.

*n*	Volatility Proxy	Bias		MSE	
		In Sample	Out of Sample	In Sample	Out of Sample
100	day	0.0204	−0.0218	0.3875	0.3979
	RV5	−0.0100	−0.0105	0.2989	0.2981
	RV15	0.0095	−0.0095	0.2804	0.2842
	RV30	0.0097	0.0092	0.2956	0.2994
200	day	−0.0196	−0.0185	0.3683	0.3759
	RV5	−0.0068	0.0057	0.2391	0.2321
	RV15	−0.0063	0.0063	0.2360	0.2221
	RV30	−0.0061	0.0067	0.2399	0.2349
500	day	0.0131	0.0128	0.2909	0.3066
	RV5	−0.0027	0.0024	0.2177	0.2185
	RV15	0.0020	0.0022	0.2129	0.2123
	RV30	0.0023	0.0021	0.2188	0.2173

**Table 4:** Bias and MSE of the conditional quantile forecasts under the parameter setting $\tau =0.05,\alpha_{0}=0.1,\;\alpha_{1}=0.2$ and $\beta_{1}=0.7$.

*n*	Volatility Proxy	Bias		MSE	
		In Sample	Out of Sample	In Sample	Out of Sample
100	day	0.0289	−0.0271	0.4325	0.4652
	RV5	0.0146	0.0141	0.3525	0.3638
	RV15	0.0125	0.0132	0.3521	0.3565
	RV30	0.0134	0.0143	0.3595	0.3653
200	day	0.0161	0.0172	0.3704	0.3637
	RV5	0.0065	0.0069	0.2627	0.2742
	RV15	0.0066	0.0063	0.2324	0.2243
	RV30	0.0065	0.0067	0.2458	0.2588
500	day	−0.0124	−0.0138	0.2620	0.2640
	RV5	0.0025	0.0021	0.1984	0.1994
	RV15	0.0026	0.0023	0.1969	0.1934
	RV30	0.0026	0.0027	0.1980	0.1930

**Table 5:** Rejection rates of the test based on $Q^{⋆}\left(K\right)$ with K=6, where $Q_{i}$ denotes the test statistic corresponding to the random weight $W_{i}$, i=1,2,3.

*d*	Volatility Proxy	n=500			n=1000			n=2000		
		$Q_{1}$	$Q_{2}$	$Q_{3}$	$Q_{1}$	$Q_{2}$	$Q_{3}$	$Q_{1}$	$Q_{2}$	$Q_{3}$
0	day	0.032	0.027	0.027	0.039	0.037	0.041	0.042	0.041	0.043
	RV5	0.046	0.043	0.045	0.048	0.046	0.048	0.050	0.051	0.051
	RV15	0.045	0.044	0.041	0.047	0.045	0.046	0.050	0.052	0.051
	RV30	0.044	0.042	0.041	0.048	0.045	0.045	0.053	0.053	0.049
0.3	day	0.076	0.069	0.072	0.214	0.208	0.192	0.520	0.512	0.520
	RV5	0.089	0.083	0.085	0.319	0.310	0.320	0.660	0.650	0.660
	RV15	0.085	0.083	0.085	0.320	0.300	0.300	0.659	0.661	0.660
	RV30	0.086	0.083	0.080	0.315	0.305	0.315	0.624	0.630	0.630
0.6	day	0.290	0.285	0.279	0.570	0.610	0.591	0.810	0.812	0.820
	RV5	0.390	0.389	0.392	0.791	0.785	0.790	0.950	0.920	0.950
	RV15	0.410	0.405	0.400	0.770	0.730	0.750	0.946	0.950	0.941
	RV30	0.400	0.390	0.400	0.781	0.795	0.780	0.950	0.935	0.940

**Table 6:** Empirical coverage errors associated with 1% and 5% VaR obtained by QMELE and QMLE.

Method	Volatility Proxy	CSI 300 Index		SSE 50 Index		CSI 500 Index	
		1%	5%	1%	5%	1%	5%
QMELE	day	0.62%	−1.02%	0.75%	0.9%	−0.18%	−1.4%
	RV5	0.21%	−0.42%	0.37%	0.1%	−0.02%	−0.38%
	RV15	0.2%	−0.53%	0.39%	0.12%	−0.09%	−0.49%
	RV30	0.25%	−0.52%	0.49%	0.36%	−0.07%	−0.51%
QMLE	day	0.54%	−0.89%	0.63%	0.70%	−0.27%	−1.10%
	RV5	0.34%	−0.57%	0.42%	0.12%	−0.03%	−0.47%
	RV15	0.17%	−0.45%	0.39%	0.14%	−0.07%	−0.40%
	RV30	0.14%	−0.45%	0.53%	0.46%	−0.10%	−0.57%

**Table 7:** The QMELE estimates and their corresponding standard errors of the parameters.

Market	Quantile	$\alpha_{0}$	$\alpha_{1}$	$\beta_{1}$
CSI 300 IndexE	1%	0.0023 (0.0003)	0.1573 (0.0195)	0.8102 (0.0448)
	5%	0.0029 (0.0006)	0.2257 (0.0313)	0.7605 (0.0613)
SSE 50 Index	1%	0.0016 (0.0005)	0.1221 (0.0167)	0.8602 (0.0829)
	5%	0.0013 (0.0005)	0.1320 (0.0155)	0.8454 (0.0605)
CSI 500 Index	1%	0.0018 (0.0009)	0.1909 (0.0556)	0.7965 (0.0929)
	5%	0.0016 (0.0009)	0.1734 (0.0589)	0.8263 (0.0847)

## Data Availability

The data that support the findings of this study are available from the corresponding author upon reasonable request.

## Appendix A. Proofs of Theorems

For convenience and without ambiguity, we set some symbols as follows:

$$z_{t}\left(\theta \right)={\left(1,x_{t-1}^{2},\ldots ,x_{t-q}^{2},h_{t-1}\left(\theta \right),\ldots ,h_{t-p}\left(\theta \right)\right)}^{\prime },\;$$

$${\tilde{z}}_{t}\left(\theta \right)={\left(1,x_{t-1}^{2},\ldots ,x_{t-q}^{2},{\tilde{h}}_{t-1}^{⋆}\left(\theta \right),\ldots ,{\tilde{h}}_{t-p}^{⋆}\left(\theta \right)\right)}^{\prime },$$

and denote

$$z_{t}=z_{t}\left(\theta_{0}\right),\;\;{\overset{˘}{z}}_{t}={\tilde{z}}_{t}\left(\theta_{0}\right),\;\;{\tilde{z}}_{t}={\tilde{z}}_{t}\left({\hat{\theta }}_{n}^{⋆}\right),$$

in which ${\hat{\theta }}_{n}^{⋆}$ is obtained from step M1.

**Proof of** **Theorem 1.** We define

$$L_{n}\left(\theta \right)=\sum_{t=1}^{n}{\tilde{h}}_{t}^{⋆-1}\rho_{\tau }\left(y_{t}-\theta^{\prime }{\tilde{z}}_{t}\right),\;{\overset{˘}{L}}_{n}\left(\theta \right)=\sum_{t=1}^{n}{\tilde{h}}_{t}^{⋆-1}\rho_{\tau }\left(y_{t}-\theta^{\prime }{\overset{˘}{z}}_{t}\right).$$

Then according to Knight [27] and Zhang et al. [22], for whatever $u\in R^{p+q+1}$, denote ${\overset{˘}{e}}_{t,\tau }=y_{t}-\theta_{\tau 0}^{\prime }{\overset{˘}{z}}_{t}$, we have

(A1) $$\begin{array}{c} L_{n}\left(\theta_{\tau 0}+n^{-1/2}u\right)-{\overset{˘}{L}}_{n}\left(\theta_{\tau 0}\right)=-L_{1n}\left(u\right)+L_{2n}\left(u\right), \end{array}$$

in which

$$L_{1n}\left(u\right)=\sum_{t=1}^{n}\psi_{\tau }\left({\overset{˘}{e}}_{t,\tau }\right){\tilde{h}}_{t}^{⋆-1}\left[{\left(\theta_{\tau 0}+n^{-1/2}u\right)}^{\prime }{\tilde{z}}_{t}-\theta_{\tau 0}^{\prime }{\overset{˘}{z}}_{t}\right],$$

$$L_{2n}\left(u\right)=\sum_{t=1}^{n}{\tilde{h}}_{t}^{⋆-1}\int_{0}^{{\left(\theta_{\tau 0}+n^{-1/2}u\right)}^{\prime }{\tilde{z}}_{t}-\theta_{\tau 0}^{\prime }{\overset{˘}{z}}_{t}}\left[I\left({\overset{˘}{e}}_{t,\tau }⩽s\right)-I\left({\overset{˘}{e}}_{t,\tau }⩽0\right)\right]ds.$$

Let $u^{\left(j\right)}$ express the (j+q+1)-th component of the vector *u*, and write $\beta_{\tau 0}^{\left(j\right)}=b_{\tau }\beta_{0j}$ for j=1,…,p. For any fixed m>0, define

$$\Theta_{n}=\Theta_{n}\left(m\right)=\left\{\theta \in \Theta :∥\theta -\theta_{0}∥⩽mn^{-1/2}\right\}.$$

We are able to verified that

(A2) $$\begin{array}{c} {\left(\theta_{\tau 0}+n^{-1/2}u\right)}^{\prime }{\tilde{z}}_{t}-\theta_{\tau 0}^{\prime }{\overset{˘}{z}}_{t}=\xi_{1nt}\left({\hat{\theta }}_{n}^{⋆}\right)+\xi_{2nt}\left({\hat{\theta }}_{n}^{⋆}\right)+\xi_{3nt}\left({\hat{\theta }}_{n}^{⋆}\right), \end{array}$$

in which

$$\xi_{1nt}\left(\theta \right)=n^{-1/2}u^{\prime }z_{t}+\sum_{j=1}^{p}\beta_{\tau 0}^{\left(j\right)}\frac{\partial h_{t-j}\left(\theta_{0}\right)}{\partial \theta^{\prime }}\left(\theta -\theta_{0}\right),$$

$$\xi_{2nt}\left(\theta \right)=\frac{1}{\sqrt{n}}\sum_{j=1}^{p}u^{\left(j\right)}\left[h_{t-j}\left(\theta \right)-h_{t-j}\right]+\sum_{j=1}^{p}\beta_{\tau 0}^{\left(j\right)}\left[h_{t-j}\left(\theta \right)-h_{t-j}-\frac{\partial h_{t-j}\left(\theta_{0}\right)}{\partial \theta^{\prime }}\left(\theta -\theta_{0}\right)\right],$$

$$\begin{array}{cc} \xi_{3nt}\left(\theta \right) & =\frac{1}{\sqrt{n}}\sum_{j=1}^{p}u^{\left(j\right)}\left[{\tilde{h}}_{t-j}^{⋆}\left(\theta \right)-h_{t-j}\left(\theta \right)\right]\\ & \;\;+\sum_{j=1}^{p}\beta_{\tau 0}^{\left(j\right)}\left\{\left[{\tilde{h}}_{t-j}^{⋆}\left(\theta \right)-h_{t-j}\left(\theta \right)\right]-\left[{\tilde{h}}_{t-j}^{⋆}\left(\theta_{0}\right)-h_{t-j}\right]\right\}. \end{array}$$

By applying the Taylor expansion, we can get

(A3) $$\begin{array}{cc} \underset{\theta \in \Theta_{n}}{\sup}\left|\xi_{2nt}\left(\theta \right)\right| & =0+\frac{1}{\sqrt{n}}\sum_{j=1}^{p}u^{\left(j\right)}\frac{\partial h_{t-j}\left(\theta_{1}^{⋆}\right)}{\partial \theta^{\prime }}\left(\theta -\theta_{0}\right)\\ & =0+0+\sum_{j=1}^{p}\beta_{\tau 0}^{\left(j\right)}\frac{1}{2!}\frac{\partial^{2}h_{t-j}\left(\theta_{2}^{⋆}\right)}{\partial \theta \partial \theta^{\prime }}{\left(\theta -\theta_{0}\right)}^{\prime }\left(\theta -\theta_{0}\right)\\ & ⩽\frac{m}{n}\sum_{j=1}^{p}\left|u^{\left(j\right)}\right|\underset{\theta \in \Theta_{n}}{\sup}∥\frac{\partial h_{t-j}\left(\theta \right)}{\partial \theta }∥+\frac{m^{2}}{2n}\sum_{j=1}^{p}\left|\beta_{\tau 0}^{\left(j\right)}\right|\underset{\theta \in \Theta_{n}}{\sup}∥\frac{\partial^{2}h_{t-j}\left(\theta \right)}{\partial \theta \partial \theta^{\prime }}∥, \end{array}$$

and from Lemma S.1 in Zheng et al. [15], it follows that

(A4) $$\begin{array}{cc} \underset{\theta \in \Theta_{n}}{\sup}\left|\xi_{3nt}\left(\theta \right)\right| & ⩽\frac{1}{\sqrt{n}}\sum_{j=1}^{p}\left[\left|u^{\left(j\right)}\right|\underset{\theta \in \Theta }{\sup}\left|{\tilde{h}}_{t-j}^{⋆}\left(\theta \right)-h_{t-j}\left(\theta \right)\right|+m\left|\beta_{\tau 0}^{\left(j\right)}\right|\underset{\theta \in \Theta }{\sup}∥\frac{\partial {\tilde{h}}_{t-j}^{⋆}\left(\theta \right)}{\partial \theta }-\frac{\partial h_{t-j}\left(\theta \right)}{\partial \theta }∥\right]\\ & ⩽n^{-1/2}C\left(m\right)\rho^{t}\zeta . \end{array}$$

Moreover, we can obtain

(A5) $$\begin{array}{cc} {\overset{˘}{e}}_{t,\tau } & =y_{t}-\theta_{\tau 0}^{\prime }{\overset{˘}{z}}_{t}\\ & =h_{t}\varepsilon_{t}-\theta_{\tau 0}^{\prime }z_{t}+\theta_{\tau 0}^{\prime }z_{t}-\theta_{\tau 0}^{\prime }{\overset{˘}{z}}_{t}\\ & =h_{t}\varepsilon_{t}-b_{\tau }h_{t}+\theta_{\tau 0}^{\prime }z_{t}-\theta_{\tau 0}^{\prime }{\overset{˘}{z}}_{t}\\ & =\left(\varepsilon_{t}-b_{\tau }\right)h_{t}+a_{t}, \end{array}$$

in which $a_{t}=\sum_{j=1}^{p}\beta_{\tau 0}^{\left(j\right)}\left[h_{t-j}-{\tilde{h}}_{t-j}^{⋆}\left(\theta_{0}\right)\right]\in F_{0}.$ With the above results, We can decompose $L_{1n}\left(u\right)$ into the following parts:

(A6) $$\begin{array}{c} L_{1n}\left(u\right)=\sum_{t=1}^{n}A_{1nt}\left({\hat{\theta }}_{n}^{⋆}\right)+\sum_{t=1}^{n}A_{2nt}\left({\hat{\theta }}_{n}^{⋆}\right)+\sum_{t=1}^{n}A_{3nt}\left({\hat{\theta }}_{n}^{⋆}\right)+\sum_{t=1}^{n}A_{4nt}\left({\hat{\theta }}_{n}^{⋆}\right), \end{array}$$

in which

$$A_{1nt}\left(\theta \right)=\psi_{\tau }\left({\overset{˘}{e}}_{t,\tau }\right){\tilde{h}}_{t}^{⋆-1}\left(\theta \right)\xi_{3nt}\left(\theta \right)+\psi_{\tau }\left({\overset{˘}{e}}_{t,\tau }\right)\left[{\tilde{h}}_{t}^{⋆-1}\left(\theta \right)-h_{t}^{-1}\left(\theta \right)\right]\left[\xi_{1nt}\left(\theta \right)+\xi_{2nt}\left(\theta \right)\right],$$

$$A_{2nt}\left(\theta \right)=\left[\psi_{\tau }\left({\overset{˘}{e}}_{t,\tau }\right)-\psi_{\tau }\left(\varepsilon_{t}-b_{\tau }\right)\right]h_{t}^{-1}\left(\theta \right)\left[\xi_{1nt}\left(\theta \right)+\xi_{2nt}\left(\theta \right)\right],$$

$$A_{3nt}\left(\theta \right)=\psi_{\tau }\left(\varepsilon_{t}-b_{\tau }\right)h_{t}^{-1}\left(\theta \right)\xi_{2nt}\left(\theta \right),\;\;\;A_{4nt}\left(\theta \right)=\psi_{\tau }\left(\varepsilon_{t}-b_{\tau }\right)h_{t}^{-1}\left(\theta \right)\xi_{1nt}\left(\theta \right).$$

Consistent with the method of Zhang et al. [22], we can prove

(A7) $$\begin{array}{c} \sum_{t=1}^{n}A_{1nt}\left({\hat{\theta }}_{n}^{⋆}\right)=o_{p}\left(1\right),\;\sum_{t=1}^{n}A_{2nt}\left({\hat{\theta }}_{n}^{⋆}\right)=o_{p}\left(1\right),\;\sum_{t=1}^{n}A_{3nt}\left({\hat{\theta }}_{n}^{⋆}\right)=o_{p}\left(1\right). \end{array}$$

and

(A8) $$\begin{array}{c} \sum_{t=1}^{n}A_{4nt}\left({\hat{\theta }}_{n}^{⋆}\right)=\sum_{t=1}^{n}\psi_{\tau }\left(\varepsilon_{t}-b_{\tau }\right)h_{t}^{-1}\xi_{1nt}\left({\hat{\theta }}_{n}^{⋆}\right)+o_{p}\left(1\right)=u^{\prime }T_{1n}+T_{2n}+o_{p}\left(1\right), \end{array}$$

in which

$$\begin{array}{c} T_{1n}=\frac{1}{\sqrt{n}}\sum_{t=1}^{n}\psi_{\tau }\left(\varepsilon_{t}-b_{\tau }\right)\frac{z_{t}}{h_{t}},\;T_{2n}=\sqrt{n}{\left({\hat{\theta }}_{n}^{⋆}-\theta_{0}\right)}^{\prime }\frac{1}{\sqrt{n}}\sum_{t=1}^{n}\psi_{\tau }\left(\varepsilon_{t}-b_{\tau }\right)\sum_{j=1}^{p}\frac{\beta_{\tau 0}^{\left(j\right)}}{h_{t}}\frac{\partial h_{t-j}\left(\theta_{0}\right)}{\partial \theta }. \end{array}$$

Then based on the framework established by Zhang et al. [22], we have

(A9) $$\begin{array}{c} L_{1n}\left(u\right)=u^{\prime }T_{1n}+T_{2n}+o_{p}\left(1\right). \end{array}$$

As for $L_{2n}\left(u\right)$, we denote $I_{t}^{⋆}\left(s\right)=I\left({\overset{˘}{e}}_{t,\tau }⩽s\right)-I\left({\overset{˘}{e}}_{t,\tau }⩽0\right)$, and rewrite $L_{2n}\left(u\right)$ as

(A10) $$\begin{array}{c} L_{2n}\left(u\right)=\sum_{t=1}^{n}B_{1nt}\left({\hat{\theta }}_{n}^{⋆}\right)+\sum_{t=1}^{n}B_{2nt}\left({\hat{\theta }}_{n}^{⋆}\right)+\sum_{t=1}^{n}B_{3nt}\left({\hat{\theta }}_{n}^{⋆}\right)+\sum_{t=1}^{n}B_{4nt}\left({\hat{\theta }}_{n}^{⋆}\right), \end{array}$$

in which

$$B_{1nt}\left(\theta \right)={\tilde{h}}_{t}^{⋆-1}\left(\theta \right)\int_{\xi_{1nt}\left(\theta \right)+\xi_{2nt}\left(\theta \right)}^{\xi_{1nt}\left(\theta \right)+\xi_{2nt}\left(\theta \right)+\xi_{3nt}\left(\theta \right)}I_{t}^{⋆}\left(s\right)ds+\left[{\tilde{h}}_{t}^{⋆-1}\left(\theta \right)-h_{t}^{-1}\left(\theta \right)\right]\int_{0}^{\xi_{1nt}\left(\theta \right)+\xi_{2nt}\left(\theta \right)}I_{t}^{⋆}\left(s\right)ds,$$

$$B_{2nt}\left(\theta \right)=h_{t}^{-1}\left(\theta \right)\int_{\xi_{1nt}\left(\theta \right)}^{\xi_{1nt}\left(\theta \right)+\xi_{2nt}\left(\theta \right)}I_{t}^{⋆}\left(s\right)ds,$$

$$B_{3nt}\left(\theta \right)=\left[h_{t}^{-1}\left(\theta \right)-h_{t}^{-1}\right]\int_{0}^{\xi_{1nt}\left(\theta \right)}I_{t}^{⋆}\left(s\right)ds,$$

$$B_{4nt}\left(\theta \right)=h_{t}^{-1}\int_{0}^{\xi_{1nt}\left(\theta \right)}I_{t}^{⋆}\left(s\right)ds.$$

Using the same proof framework as Zhang et al. [22], it can be shown that

(A11) $$\begin{array}{c} \sum_{t=1}^{n}B_{1nt}\left({\hat{\theta }}_{n}^{⋆}\right)=o_{p}\left(1\right),\;\sum_{t=1}^{n}B_{2nt}\left({\hat{\theta }}_{n}^{⋆}\right)=o_{p}\left(1\right),\;\sum_{t=1}^{n}B_{3nt}\left({\hat{\theta }}_{n}^{⋆}\right)=o_{p}\left(1\right), \end{array}$$

and

(A12) $$\begin{array}{cc} \sum_{t=1}^{n}B_{4nt}^{⋆}\left(\theta \right) & =\sum_{t=1}^{n}h_{t}^{-1}\int_{0}^{\xi_{1nt}\left(\theta \right)}f\left(b_{\tau }-h_{t}^{-1}a_{t}\right)h_{t}^{-1}sds+R_{1n}\left(\theta \right)\\ & =\frac{1}{2}f\left(b_{\tau }\right)\sum_{t=1}^{n}h_{t}^{-2}\xi_{1nt}^{2}\left(\theta \right)+R_{2n}\left(\theta \right)+R_{1n}\left(\theta \right), \end{array}$$

in which

$$\begin{array}{c} R_{1n}\left(\theta \right)=\frac{1}{2}\sum_{t=1}^{n}h_{t}^{-3}\int_{0}^{\xi_{1nt}\left(\theta \right)}\dot{f}\left(b_{\tau ,t}^{⋆}\left(s\right)\right)s^{2}ds, \end{array}$$

with $b_{\tau }-h_{t}^{-1}a_{t}<b_{\tau ,t}^{⋆}\left(s\right)<b_{\tau }-h_{t}^{-1}a_{t}+h_{t}^{-1}s$, and

$$\begin{array}{c} R_{2n}\left(\theta \right)=\frac{1}{2}\sum_{t=1}^{n}h_{t}^{-2}\xi_{1nt}^{2}\left(\theta \right)\left[f\left(b_{\tau }-h_{t}^{-1}a_{t}\right)-f\left(b_{\tau }\right)\right]. \end{array}$$

Moreover, it holds that

$$\begin{array}{c} R_{1n}\left({\hat{\theta }}_{n}^{⋆}\right)=o_{p}\left(1\right),\;R_{2n}\left({\hat{\theta }}_{n}^{⋆}\right)=o_{p}\left(1\right). \end{array}$$

Since ${\left(a^{\prime }b\right)}^{2}=\left(a^{\prime }b\right){\left(a^{\prime }b\right)}^{\prime }=a^{\prime }bb^{\prime }a$. the ergodic theorem implies that

(A13) $$\begin{array}{cc} L_{2n}\left(u\right)= & \frac{1}{2}f\left(b_{\tau }\right)\sum_{t=1}^{n}h_{t}^{-2}\xi_{1nt}^{2}\left({\hat{\theta }}_{n}^{⋆}\right)+o_{p}\left(1\right)\\ = & \frac{1}{2}f\left(b_{\tau }\right)\sum_{t=1}^{n}h_{t}^{-2}{\left[\frac{1}{\sqrt{n}}u^{\prime }z_{t}+\sum_{j=1}^{p}\beta_{\tau 0}^{\left(j\right)}\frac{\partial h_{t-j}\left(\theta_{0}\right)}{\partial \theta^{\prime }}\left({\hat{\theta }}_{n}^{⋆}-\theta_{0}\right)\right]}^{2}+o_{p}\left(1\right)\\ = & \frac{1}{2}f\left(b_{\tau }\right)\sum_{t=1}^{n}h_{t}^{-2}\left[\frac{1}{n}u^{\prime }z_{t}z_{t}^{\prime }u+\frac{2}{\sqrt{n}}u^{\prime }z_{t}\sum_{j=1}^{p}\beta_{\tau 0}^{\left(j\right)}\frac{\partial h_{t-j}\left(\theta_{0}\right)}{\partial \theta^{\prime }}\left({\hat{\theta }}_{n}^{⋆}-\theta_{0}\right)\right.\\ & \;\;+\left.{\left(\sum_{j=1}^{p}\beta_{\tau 0}^{\left(j\right)}\frac{\partial h_{t-j}\left(\theta_{0}\right)}{\partial \theta^{\prime }}\left({\hat{\theta }}_{n}^{⋆}-\theta_{0}\right)\right)}^{2}\right]+o_{p}\left(1\right)\\ = & \frac{1}{2}f\left(b_{\tau }\right)u^{\prime }\Omega u+b_{\tau }f\left(b_{\tau }\right)u^{\prime }\Gamma \sqrt{n}\left({\hat{\theta }}_{n}^{⋆}-\theta_{0}\right)+T_{3n}+o_{p}\left(1\right), \end{array}$$

in which

$$\begin{array}{c} T_{3n}=\frac{1}{2}f\left(b_{\tau }\right){\left({\hat{\theta }}_{n}^{⋆}-\theta_{0}\right)}^{\prime }\sum_{t=1}^{n}\sum_{j_{1}=1}^{p}\sum_{j_{2}=1}^{p}\beta_{\tau 0}^{\left(j_{1}\right)}\beta_{\tau 0}^{\left(j_{2}\right)}\frac{1}{h_{t}^{2}}\frac{\partial h_{t-j_{1}}\left(\theta_{0}\right)}{\partial \theta }\frac{\partial h_{t-j_{2}}\left(\theta_{0}\right)}{\partial \theta^{\prime }}\left({\tilde{\theta }}_{n}-\theta_{0}\right). \end{array}$$

A combination of (A1), (A9) and (A13) gives that

$$\begin{array}{cc} L_{n}\left(\theta_{\tau 0}+n^{-1/2}u\right)-{\overset{˘}{L}}_{n}\left(\theta_{\tau 0}\right)= & -L_{1n}\left(u\right)+L_{2n}\left(u\right)\\ & -u^{\prime }\left[T_{1n}-b_{\tau }f\left(b_{\tau }\right)\Gamma \sqrt{n}\left({\hat{\theta }}_{n}^{⋆}-\theta_{0}\right)\right]+\frac{1}{2}f\left(b_{\tau }\right)u^{\prime }\Omega u\\ & -T_{2n}+T_{3n}+o_{p}\left(1\right). \end{array}$$

Following the same methodology as Zhang et al. [22], we have

$$\begin{array}{c} \sqrt{n}\left({\tilde{\theta }}_{n}^{⋆}-\theta_{0}^{⋆}\right)=-\frac{J^{⋆-1}}{\sqrt{n}}\sum_{t=1}^{n}\frac{1-\varepsilon_{t}^{⋆}}{2h_{t}^{⋆}\left(\theta_{0}^{⋆}\right)}\frac{\partial h_{t}^{⋆}\left(\theta_{0}^{⋆}\right)}{\partial \theta^{⋆}}+o_{p}\left(1\right), \end{array}$$

in which $E(\varepsilon_{t}^{⋆})=1$, and the symbol “⋆” indicates the incorporation of high-frequency information into the parameters. As a result, we obtain

$$\begin{array}{c} \sqrt{n}\left(\hat{M}{\hat{\theta }}_{n}^{⋆}-M\theta_{0}\right)=-\frac{J^{⋆-1}}{\sqrt{n}}\sum_{t=1}^{n}\frac{1-\varepsilon_{t}^{⋆}}{2h_{t}^{⋆}\left(\theta_{0}^{⋆}\right)}\frac{\partial h_{t}^{⋆}\left(\theta_{0}^{⋆}\right)}{\partial \theta^{⋆}}+o_{p}\left(1\right), \end{array}$$

in which

$$M=\mathrm{diag}\left\{\underset{q+1}{\underset{︸}{1/\mu^{2},\ldots ,1/\mu^{2}}},\underset{p}{\underset{︸}{1,\ldots ,1}}\right\},\;\hat{M}=\mathrm{diag}\left\{\underset{q+1}{\underset{︸}{1/{\hat{\mu }}^{2},\ldots ,1/{\hat{\mu }}^{2}}},\underset{p}{\underset{︸}{1,\ldots ,1}}\right\},$$

with $\hat{\mu }$ constituting a consistent estimator of μ. Then, it follows that

(A14) $$\begin{array}{c} \sqrt{n}\left({\hat{\theta }}_{n}^{⋆}-\theta_{0}\right)=M^{-1}\left(-\frac{J^{⋆-1}}{\sqrt{n}}\sum_{t=1}^{n}\frac{1-\varepsilon_{t}^{⋆}}{2h_{t}^{⋆}\left(\theta_{0}^{⋆}\right)}\frac{\partial h_{t}^{⋆}\left(\theta_{0}^{⋆}\right)}{\partial \theta^{⋆}}\right)+o_{p}\left(1\right). \end{array}$$

Noting the fact that

$$\begin{array}{ccc} \;{\hat{\theta }}_{\tau n}^{⋆}-\theta_{\tau 0}= & \underset{\theta }{\arg\min}\left[L_{n}\left(\theta \right)-{\overset{˘}{L}}_{n}\left(\theta_{\tau 0}\right)\right] & \\ = & \underset{\theta }{\arg\min}[-\sqrt{n}{\left(\theta -\theta_{\tau 0}\right)}^{\prime }\left(T_{1n}-b_{\tau }f\left(b_{\tau }\right)\Gamma \sqrt{n}\left({\hat{\theta }}_{n}^{⋆}-\theta_{0}\right)\right) & \\ & \;+\frac{1}{2}f\left(b_{\tau }\right)\sqrt{n}{\left(\theta -\theta_{\tau 0}\right)}^{\prime }\Omega \sqrt{n}\left(\theta -\theta_{\tau 0}\right)] \end{array}$$

and

$$\begin{array}{c} -\sqrt{n}\left[T_{1n}-b_{\tau }f\left(b_{\tau }\right)\Gamma \sqrt{n}\left({\hat{\theta }}_{n}^{⋆}-\theta_{0}\right)\right]+\sqrt{n}f\left(b_{\tau }\right)\Omega \sqrt{n}\left(\theta -\theta_{\tau 0}\right)+o_{p}\left(1\right)=0, \end{array}$$

we can get

$$\begin{array}{cc} \sqrt{n}\left({\hat{\theta }}_{\tau n}^{⋆}-\theta_{\tau 0}\right)= & \Omega^{-1}\left(\frac{T_{1n}-b_{\tau }f\left(b_{\tau }\right)\Gamma \sqrt{n}\left({\hat{\theta }}_{n}^{⋆}-\theta_{0}\right)}{f(b_{\tau })}\right)\\ = & \frac{\Omega^{-1}}{f(b_{\tau })}T_{1n}-b_{\tau }\Omega^{-1}\Gamma \sqrt{n}\left({\hat{\theta }}_{n}^{⋆}-\theta_{0}\right)+o_{p}\left(1\right). \end{array}$$

Based on Corollary 2 in Knight [27], as well as the Liapounov’s central limit theorem, we have

(A15) $$\begin{array}{c} \sqrt{n}\left({\hat{\theta }}_{\tau n}^{⋆}-\theta_{\tau 0}\right)=\frac{\Omega^{-1}}{f\left(b_{\tau }\right)}T_{1n}-b_{\tau }\Omega^{-1}\Gamma \sqrt{n}\left({\hat{\theta }}_{n}^{⋆}-\theta_{0}\right)+o_{p}\left(1\right)\overset{d}{⟶}N\left(0,\Sigma_{2}^{⋆}\right), \end{array}$$

in which $T_{1n}=\frac{1}{\sqrt{n}}\sum_{t=1}^{n}\psi_{\tau }\left(\varepsilon_{t}-b_{\tau }\right)\frac{z_{t}}{h_{t}}$. Furthermore, leveraging the ergodic theorem, we are able to derive $\Sigma_{2}^{⋆}$ as

$$\begin{array}{cc} \Sigma_{2}^{⋆}= & E\left[\sqrt{n}\left({\hat{\theta }}_{\tau n}^{⋆}-\theta_{\tau 0}\right)\sqrt{n}{\left({\hat{\theta }}_{\tau n}^{⋆}-\theta_{\tau 0}\right)}^{\prime }\right]\\ = & E\left[\frac{1}{f^{2}\left(b_{\tau }\right)}\Omega^{-1}\left(\frac{1}{\sqrt{n}}\sum_{t=1}^{n}\psi_{\tau }\left(\varepsilon_{t}-b_{\tau }\right)\frac{z_{t}}{h_{t}}\right){\left(\frac{1}{\sqrt{n}}\sum_{t=1}^{n}\psi_{\tau }\left(\varepsilon_{t}-b_{\tau }\right)\frac{z_{t}}{h_{t}}\right)}^{\prime }\Omega^{-1}\right]\\ & -E\left[\frac{b_{\tau }}{f\left(b_{\tau }\right)}\Omega^{-1}\left(\frac{1}{\sqrt{n}}\sum_{t=1}^{n}\psi_{\tau }\left(\varepsilon_{t}-b_{\tau }\right)\frac{z_{t}}{h_{t}}\right)\sqrt{n}{\left({\hat{\theta }}_{n}^{⋆}-\theta_{0}\right)}^{\prime }\Gamma^{\prime }\Omega^{-1}\right]\\ & -E\left[\frac{b_{\tau }}{f\left(b_{\tau }\right)}\Omega^{-1}\Gamma \sqrt{n}\left({\hat{\theta }}_{n}^{⋆}-\theta_{0}\right){\left(\frac{1}{\sqrt{n}}\sum_{t=1}^{n}\psi_{\tau }\left(\varepsilon_{t}-b_{\tau }\right)\frac{z_{t}}{h_{t}}\right)}^{\prime }\Omega^{-1}\right]\\ & +E\left[b_{\tau }^{2}\Omega^{-1}\Gamma \sqrt{n}\left({\hat{\theta }}_{n}^{⋆}-\theta_{0}\right)\sqrt{n}{\left({\hat{\theta }}_{n}^{⋆}-\theta_{0}\right)}^{\prime }\Gamma^{-1}\Omega^{-1}\right]\\ = & \frac{1}{f^{2}\left(b_{\tau }\right)}\Omega^{-1}\left(\tau -\tau^{2}\right)\Omega \Omega^{-1}+\frac{\kappa_{1}^{⋆}b_{\tau }}{f\left(b_{\tau }\right)}\Omega^{-1}H^{⋆}J^{⋆-1}M^{-1}\Gamma^{\prime }\Omega^{-1}\\ & +\frac{\kappa_{1}^{⋆}b_{\tau }}{f\left(b_{\tau }\right)}\Omega^{-1}\Gamma M^{-1}J^{⋆-1}H^{⋆\prime }\Omega^{-1}+\kappa_{2}^{⋆}b_{\tau }^{2}\Omega^{-1}\Gamma M^{-1}J^{⋆-1}M^{-1}\Gamma^{\prime }\Omega^{-1}\\ = & \Omega^{-1}\left[\frac{\tau -\tau^{2}}{f^{2}\left(b_{\tau }\right)}\Omega +\frac{\kappa_{1}^{⋆}b_{\tau }}{f\left(b_{\tau }\right)}\left(H^{⋆}J^{⋆-1}M^{-1}\Gamma^{\prime }+\Gamma M^{-1}J^{⋆-1}H^{⋆\prime }\right)+\kappa_{2}^{⋆}b_{\tau }^{2}\Gamma M^{-1}J^{⋆-1}M^{-1}\Gamma^{\prime }\right]\Omega^{-1}, \end{array}$$

in which $\kappa_{1}^{⋆}=\left(\frac{1}{2}E\left[\epsilon_{t}^{⋆}I\left(\eta_{t}<Q_{\tau ,\eta }\right)\right]-\tau \right)$ and $\kappa_{2}^{⋆}=4\mathrm{Var}\left(\epsilon_{t}^{*}\right)$. Therefore, the proof of the theorem is hereby completed □

**Proof of** **Theorem 2.** To simplify the notation, we write

$$L_{n}^{*}\left(\theta \right)=\sum_{t=1}^{n}\omega_{t}{\tilde{h}}_{t}^{⋆-1}\rho_{\tau }\left(y_{t}-\theta^{\prime }{\tilde{z}}_{t}^{*}\right),\;{\overset{˘}{L}}_{n}^{*}\left(\theta \right)=\sum_{t=1}^{n}\omega_{t}{\tilde{h}}_{t}^{⋆-1}\rho_{\tau }\left(y_{t}-\right.\left.\theta^{\prime }{\overset{˘}{z}}_{t}\right),$$

then for each $u\in R^{p+q+1}$, by the same argument as in the proof of Theorem 1, we have

(A16) $$\begin{array}{c} L_{n}^{*}\left(\theta_{\tau 0}+n^{-1/2}u\right)-{\overset{˘}{L}}_{n}^{*}\left(\theta_{\tau 0}\right)=-L_{1n}^{*}\left(u\right)+L_{2n}^{*}\left(u\right), \end{array}$$

in which

$$L_{1n}^{*}\left(u\right)=\sum_{t=1}^{n}\omega_{t}\psi_{\tau }\left({\overset{˘}{e}}_{t,\tau }\right){\tilde{h}}_{t}^{⋆-1}\left[{\left(\theta_{\tau 0}+n^{-1/2}u\right)}^{\prime }{\tilde{z}}_{t}^{*}-\theta_{\tau 0}^{\prime }{\overset{˘}{z}}_{t}\right],$$

$$L_{2n}^{*}\left(u\right)=\sum_{t=1}^{n}\omega_{t}{\tilde{h}}_{t}^{⋆-1}\int_{0}^{{\left(\theta_{\tau 0}+n^{-1/2}u\right)}^{\prime }{\tilde{z}}_{t}^{*}-\theta_{\tau 0}^{\prime }{\overset{˘}{z}}_{t}}\left[I\left({\overset{˘}{e}}_{t,\tau }⩽s\right)-I\left({\overset{˘}{e}}_{t,\tau }⩽0\right)\right]ds,$$

and

$$\begin{array}{c} {\left(\theta_{\tau 0}+n^{-1/2}u\right)}^{\prime }{\tilde{z}}_{t}^{*}-\theta_{\tau 0}^{\prime }{\overset{˘}{z}}_{t}=\xi_{1nt}\left({\hat{\theta }}_{n}^{*}\right)+\xi_{2nt}\left({\hat{\theta }}_{n}^{*}\right)+\xi_{3nt}\left({\hat{\theta }}_{n}^{*}\right). \end{array}$$

Following the same reasoning as in the proof of Theorem 1, it follows that

(A17) $$\begin{array}{cc} \sqrt{n}\left({\hat{\theta }}_{n}^{*}-{\hat{\theta }}_{n}^{⋆}\right) & =\sqrt{n}\left({\hat{\theta }}_{n}^{*}-\theta_{0}\right)-\sqrt{n}\left({\hat{\theta }}_{n}^{⋆}-\theta_{0}\right)\\ & =M^{-1}\left(-\frac{J^{⋆-1}}{\sqrt{n}}\sum_{t=1}^{n}\left(\omega_{t}-1\right)\left(1-\frac{H_{t}}{\sqrt{h_{t}^{⋆}\left(\theta_{0}^{⋆}\right)}}\right)\frac{1}{2h_{t}^{⋆}\left(\theta_{0}^{*}\right)}\frac{\partial h_{t}^{⋆}\left(\theta_{0}^{⋆}\right)}{\partial \theta^{⋆}}\right)+o_{p}\left(1\right), \end{array}$$

and

(A18) $$\begin{array}{c} \sqrt{n}\left({\hat{\theta }}_{n}^{*}-\theta_{0}\right)\sqrt{n}\left({\hat{\theta }}_{n}^{*}-{\hat{\theta }}_{n}^{⋆}\right)+\sqrt{n}\left({\hat{\theta }}_{n}^{⋆}-\theta_{0}\right)=O_{p}\left(1\right). \end{array}$$

To improve clarity, the functions $A_{\mathrm{int}\;}$ for 1⩽i⩽4 in the proof of Theorem 1 are rewritten as follows:

$$A_{1nt}\left(\theta_{1},\theta_{2}\right)=\psi_{\tau }\left({\overset{˘}{e}}_{t,\tau }\right){\tilde{h}}_{t}^{⋆-1}\left(\theta_{1}\right)\xi_{3nt}\left(\theta_{2}\right)+\psi_{\tau }\left({\overset{˘}{e}}_{t,\tau }\right)\left[{\tilde{h}}_{t}^{⋆-1}\left(\theta_{1}\right)-h_{t}^{-1}\left(\theta_{1}\right)\right]\left[\xi_{1nt}\left(\theta_{2}\right)+\xi_{2nt}\left(\theta_{2}\right)\right],$$

$$A_{2nt}\left(\theta_{1},\theta_{2}\right)=\left[\psi_{\tau }\left({\overset{˘}{e}}_{t,\tau }\right)-\psi_{\tau }\left(\varepsilon_{t}-b_{\tau }\right)\right]h_{t}^{-1}\left(\theta_{1}\right)\left[\xi_{1nt}\left(\theta_{2}\right)+\xi_{2nt}\left(\theta_{2}\right)\right],$$

$$A_{3nt}\left(\theta_{1},\theta_{2}\right)=\psi_{\tau }\left(\varepsilon_{t}-b_{\tau }\right)h_{t}^{-1}\left(\theta_{1}\right)\xi_{2nt}\left(\theta_{2}\right),$$

$$A_{4nt}\left(\theta_{1},\theta_{2}\right)=\psi_{\tau }\left(\varepsilon_{t}-b_{\tau }\right)h_{t}^{-1}\left(\theta_{1}\right)\xi_{1nt}\left(\theta_{2}\right),$$

and similarly, we rewrite $B_{int}$ for 1⩽i⩽3 as

$$\begin{array}{cc} B_{1nt}\left(\theta_{1},\theta_{2}\right)= & {\tilde{h}}_{t}^{⋆-1}\left(\theta_{1}\right)\int_{\xi_{1nt}\left(\theta_{2}\right)+\xi_{2nt}\left(\theta_{2}\right)}^{\xi_{1nt}\left(\theta_{2}\right)+\xi_{2nt}\left(\theta_{2}\right)+\xi_{3nt}\left(\theta_{2}\right)}I_{t}^{⋆}\left(s\right)ds\\ & +\left[{\tilde{h}}_{t}^{⋆-1}\left(\theta_{1}\right)-h_{t}^{-1}\left(\theta_{1}\right)\right]\int_{0}^{\xi_{1nt}\left(\theta_{2}\right)+\xi_{2nt}\left(\theta_{2}\right)}I_{t}^{⋆}\left(s\right)ds\\ B_{2nt}\left(\theta_{1},\theta_{2}\right)= & h_{t}^{-1}\left(\theta_{1}\right)\int_{\xi_{1nt}\left(\theta_{2}\right)}^{\xi_{1nt}\left(\theta_{2}\right)+\xi_{2nt}\left(\theta_{2}\right)}I_{t}^{⋆}\left(s\right)ds,\\ B_{3nt}\left(\theta_{1},\theta_{2}\right)= & \left[h_{t}^{-1}\left(\theta_{1}\right)-h_{t}^{-1}\right]\int_{0}^{\xi_{1nt}\left(\theta_{2}\right)}I_{t}^{⋆}\left(s\right)ds, \end{array}$$

Besides, the function $B_{4nt}\left(\cdot \right)$ is defined in a manner consistent with the proof of Theorem 1. For 1≤i≤3, by applying the approach proposed by Zhang et al. [22], we can obtain

$$\begin{array}{c} \sum_{t=1}^{n}\omega_{t}A_{\mathrm{int}\;}\left({\hat{\theta }}_{n}^{⋆},{\hat{\theta }}_{n}^{*}\right)=o_{p}\left(1\right), \end{array}$$

and for i=4, we have

$$\begin{array}{c} \sum_{t=1}^{n}\omega_{t}A_{4nt}\left({\hat{\theta }}_{n}^{⋆},{\hat{\theta }}_{n}^{*}\right)=\sum_{t=1}^{n}\omega_{t}\psi_{\tau }\left(\varepsilon_{t}-b_{\tau }\right)h_{t}^{-1}\xi_{1nt}\left({\hat{\theta }}_{n}^{*}\right)+o_{p}^{*}\left(1\right)=u^{\prime }T_{1n}^{*}+T_{2n}^{*}+o_{p}\left(1\right), \end{array}$$

where $T_{1n}^{*}=n^{-1/2}\sum_{t=1}^{n}\omega_{t}\psi_{\tau }\left(\varepsilon_{t}-b_{\tau }\right)z_{t}/h_{t}$, as well as

$$\begin{array}{c} T_{2n}^{*}=\sqrt{n}{\left({\hat{\theta }}_{n}^{*}-\theta_{0}\right)}^{\prime }\frac{1}{\sqrt{n}}\sum_{t=1}^{n}\omega_{t}\psi_{\tau }\left(\varepsilon_{t}-b_{\tau }\right)\sum_{j=1}^{p}\frac{\beta_{\tau 0}^{\left(j\right)}}{h_{t}}\frac{\partial h_{t-j}\left(\theta_{0}\right)}{\partial \theta }, \end{array}$$

in which $\beta_{\tau 0}^{\left(j\right)}=b_{\tau }\beta_{0j},j=1,\ldots ,p$. Then, it follows that

(A19) $$\begin{array}{cc} L_{1n}^{*}\left(u\right) & =\sum_{t=1}^{n}\omega_{t}A_{1nt}\left({\hat{\theta }}_{n}^{⋆},{\hat{\theta }}_{n}^{*}\right)+\sum_{t=1}^{n}\omega_{t}A_{2nt}\left({\hat{\theta }}_{n}^{⋆},{\hat{\theta }}_{n}^{*}\right)+\sum_{t=1}^{n}\omega_{t}A_{3nt}\left({\hat{\theta }}_{n}^{⋆},{\hat{\theta }}_{n}^{*}\right)+\sum_{t=1}^{n}\omega_{t}A_{4nt}\left({\hat{\theta }}_{n},{\tilde{\theta }}_{n}^{*}\right)\\ & =u^{\prime }T_{1n}^{*}+T_{2n}^{*}+o_{p}^{*}\left(1\right), \end{array}$$

and

$$\begin{array}{c} \sum_{t=1}^{n}\left(\omega_{t}-1\right)B_{\mathrm{int}}\left({\hat{\theta }}_{n}^{⋆},\;{\hat{\theta }}_{n}^{*}\right)=o_{p}\left(1\right),\;1⩽i⩽3,\;\sum_{t=1}^{n}\left(\omega_{t}-1\right)B_{4nt}\left({\hat{\theta }}_{n}^{*}\right)=o_{p}\left(1\right), \end{array}$$

which indicate that

$$\begin{array}{c} L_{2n}^{*}\left(u\right)=\sum_{t=1}^{n}B_{1nt}\left({\hat{\theta }}_{n}^{⋆},{\hat{\theta }}_{n}^{*}\right)+\sum_{t=1}^{n}B_{2nt}\left({\hat{\theta }}_{n}^{⋆},{\hat{\theta }}_{n}^{*}\right)+\sum_{t=1}^{n}B_{3nt}\left({\hat{\theta }}_{n}^{⋆},{\hat{\theta }}_{n}^{*}\right)+\sum_{t=1}^{n}B_{4nt}\left({\hat{\theta }}_{n}^{*}\right)+o_{p}\left(1\right). \end{array}$$

Hence, following the same way used to derive (A13), it can be further shown that

(A20) $$\begin{array}{c} L_{2n}^{*}\left(u\right)=\frac{1}{2}f\left(b_{\tau }\right)u^{\prime }\Omega u+b_{\tau }f\left(b_{\tau }\right)u^{\prime }\Gamma \sqrt{n}\left({\hat{\theta }}_{n}^{*}-\theta_{0}\right)+T_{3n}^{*}+o_{p}\left(1\right), \end{array}$$

where

$$\begin{array}{c} T_{3n}^{*}=\frac{1}{2}f\left(b_{\tau }\right){\left({\hat{\theta }}_{n}^{*}-\theta_{0}\right)}^{\prime }\sum_{t=1}^{n}\sum_{j_{1}=1}^{p}\sum_{j_{2}=1}^{p}\beta_{\tau 0}^{\left(j_{1}\right)}\beta_{\tau 0}^{\left(j_{2}\right)}\frac{1}{h_{t}^{2}}\frac{\partial h_{t-j_{1}}\left(\theta_{0}\right)}{\partial \theta }\frac{\partial h_{t-j_{2}}\left(\theta_{0}\right)}{\partial \theta^{\prime }}\left({\hat{\theta }}_{n}^{*}-\theta_{0}\right). \end{array}$$

Therefore, by combining (A16), (A19) and (A20), we obtain

$$\begin{array}{cc} L_{n}^{*}\left(\theta_{\tau 0}+n^{-1/2}u\right)-{\overset{˘}{L}}_{n}^{*}\left(\theta_{\tau 0}\right)= & -u^{\prime }\left[T_{1n}^{*}-b_{\tau }f\left(b_{\tau }\right)\Gamma \sqrt{n}\left({\hat{\theta }}_{n}^{*}-\theta_{0}\right)\right]+\frac{1}{2}f\left(b_{\tau }\right)u^{\prime }\Omega u\\ & -T_{2n}^{*}+T_{3n}^{*}+o_{p}\left(1\right), \end{array}$$

in which $T_{1n}^{*}=n^{-1/2}\sum_{t=1}^{n}\omega_{t}\psi_{\tau }\left(\varepsilon_{t}-b_{\tau }\right)z_{t}/h_{t}$. Let $X_{t}=n^{-1/2}\left(\omega_{t}-1\right)\psi_{\tau }\left(\varepsilon_{t}-b_{\tau }\right)z_{t}/h_{t}$, it is easy to get that

$$T_{1n}^{*}-T_{1n}=\sum_{t=1}^{n}X_{t}.$$

Then, similar to Zhang et al. [22], by the Lindeberg’s central limit theorem and the Cramér-Wold device, it holds that

$$\begin{array}{c} T_{1n}^{*}-T_{1n}=\sum_{t=1}^{n}X_{t}\overset{d}{⟶}N\left(0,\tau (1-\tau )\Omega \right),\;n\to \infty , \end{array}$$

therefore, it can be concluded that

(A21) $$\begin{array}{c} \sqrt{n}\left({\hat{\theta }}_{\tau n}^{*}-\theta_{\tau 0}\right)=\frac{\Omega^{-1}}{f\left(b_{\tau }\right)}T_{1n}^{*}-b_{\tau }\Omega^{-1}\Gamma \sqrt{n}\left({\hat{\theta }}_{n}^{*}-\theta_{0}\right)+o_{p}\left(1\right). \end{array}$$

From the above results and (A15), the Bahadur representation of the mixed bootstrap estimator ${\hat{\theta }}_{\tau n}^{*}$ can be derived as

$$\begin{array}{cc} \sqrt{n}\left({\hat{\theta }}_{\tau n}^{*}-{\hat{\theta }}_{\tau n}^{⋆}\right) & =\frac{\Omega^{-1}}{f\left(b_{\tau }\right)}\left(T_{1n}^{*}-T_{1n}\right)\\ & +\frac{b_{\tau }\Omega^{-1}\Gamma J^{⋆-1}}{\sqrt{n}}M^{-1}\left(\sum_{t=1}^{n}\left(\omega_{t}-1\right)\left(1-\frac{H_{t}}{\sqrt{h_{t}^{⋆}\left(\theta_{0}^{⋆}\right)}}\right)\frac{1}{2h_{t}^{⋆}\left(\theta_{0}^{*}\right)}\frac{\partial h_{t}^{⋆}\left(\theta_{0}^{⋆}\right)}{\partial \theta^{⋆}}\right)+o_{p}\left(1\right). \end{array}$$

At last, we can verify the Liapounov’s condition, and complete the proof of the theorem by applying the Lindeberg central limit theorem and the Cramér–Wold device. □

**Proof of** **Theorem 3.** First, we can infer that

(A22) $$\begin{array}{c} \frac{1}{\sqrt{n}}\sum_{t=k+1}^{n}\psi_{\tau }\left({\hat{\varepsilon }}_{t,\tau }^{⋆}\right)\left|{\hat{\varepsilon }}_{t-k,\tau }^{⋆}\right|=\frac{1}{\sqrt{n}}\sum_{t=k+1}^{n}\psi_{\tau }\left(\varepsilon_{t,\tau }\right)\left|\varepsilon_{t-k,\tau }\right|+\sum_{t=k+1}^{n}E_{1nt}+\sum_{t=k+1}^{n}E_{2nt}+\sum_{t=k+1}^{n}E_{3nt}, \end{array}$$

in which

$$E_{1nt}=n^{-1/2}\left[\psi_{\tau }\left({\hat{\varepsilon }}_{t,\tau }^{⋆}\right)-\psi_{\tau }\left(\varepsilon_{t,\tau }\right)\right]\left|\varepsilon_{t-k,\tau }\right|,$$

$$E_{2nt}=n^{-1/2}\psi_{\tau }\left(\varepsilon_{t,\tau }\right)\left(\left|{\hat{\varepsilon }}_{t-k,\tau }^{⋆}\right|-\left|\varepsilon_{t-k,\tau }\right|\right),$$

$$E_{3nt}=n^{-1/2}\left[\psi_{\tau }\left({\hat{\varepsilon }}_{t,\tau }^{⋆}\right)-\psi_{\tau }\left(\varepsilon_{t,\tau }\right)\right]\left(\left|{\hat{\varepsilon }}_{t-k,\tau }^{⋆}\right|-\left|\varepsilon_{t-k,\tau }\right|\right).$$

Following Zhang et al. [22], and using the same argument, we can show that

(A23) $$\begin{array}{c} \sum_{t=k+1}^{n}E_{1nt}=-f\left(b_{\tau }\right)\left[d_{1k}^{\prime }\sqrt{n}\left({\hat{\theta }}_{\tau n}^{⋆}-\theta_{\tau 0}\right)+b_{\tau }d_{2k}^{\prime }\sqrt{n}\left({\hat{\theta }}_{n}^{⋆}-\theta_{0}\right)\right]+o_{p}\left(1\right), \end{array}$$

where

$$d_{1k}=E\left(h_{t}^{-1}\left|\varepsilon_{t-k,\tau }\right|z_{t}\right),\;d_{2k}=E\left(h_{t}^{-1}\left|\varepsilon_{t-k,\tau }\right|\sum_{j=1}^{p}\beta_{0j}\partial h_{t-j}\left(\theta_{0}\right)/\partial \theta \right),$$

as well as

(A24) $$\begin{array}{c} \sum_{t=k+1}^{n}E_{2nt}=o_{p}\left(1\right),\;\sum_{t=k+1}^{n}E_{3nt}=o_{p}\left(1\right). \end{array}$$

Hence, by combining (A22)–(A24), we obtain that

(A25) $$\begin{array}{cc} \frac{1}{\sqrt{n}}\sum_{t=k+1}^{n}\psi_{\tau }\left({\hat{\varepsilon }}_{t,\tau }^{⋆}\right)\left|{\hat{\varepsilon }}_{t-k,\tau }^{⋆}\right|= & \frac{1}{\sqrt{n}}\sum_{t=k+1}^{n}\psi_{\tau }\left(\varepsilon_{t,\tau }\right)\left|\varepsilon_{t-k,\tau }\right|\\ & -f\left(b_{\tau }\right)\left[d_{1k}^{\prime }\sqrt{n}\left({\hat{\theta }}_{\tau n}^{⋆}-\theta_{\tau 0}\right)+b_{\tau }d_{2k}^{\prime }\sqrt{n}\left({\hat{\theta }}_{n}^{⋆}-\theta_{0}\right)\right]+o_{p}\left(1\right). \end{array}$$

Finally, an application of the law of large numbers yields that

$$\begin{array}{c} \left|{\hat{\mu }}_{a,\tau }-\mu_{a,\tau }\right|=\left|\frac{1}{n}\sum_{t=1}^{n}\left(\left|{\hat{\varepsilon }}_{t,\tau }^{⋆}\right|-\left|\varepsilon_{t,\tau }\right|\right)\right|+o_{p}\left(1\right)⩽\frac{1}{n}\sum_{t=1}^{n}\left|{\hat{\varepsilon }}_{t,\tau }^{⋆}-\varepsilon_{t,\tau }\right|+o_{p}\left(1\right)=o_{p}\left(1\right), \end{array}$$

which leads to the conclusion that

$$\begin{array}{cc} {\hat{\sigma }}_{a,\tau }^{2} & =\frac{1}{n}\sum_{t=1}^{n}{\left(\left|{\hat{\varepsilon }}_{t,\tau }^{⋆}\right|-{\hat{\mu }}_{a,\tau }\right)}^{2}\\ & =\frac{1}{n}\sum_{t=1}^{n}{\hat{\varepsilon }}_{t,\tau }^{⋆2}-\mu_{a,\tau }^{2}+o_{p}\left(1\right)\\ & =\frac{1}{n}\sum_{t=1}^{n}\left({\hat{\varepsilon }}_{t,\tau }^{⋆2}-\varepsilon_{t,\tau }^{2}\right)+\sigma_{a,\tau }^{2}+o_{p}\left(1\right)\\ & =\sigma_{a,\tau }^{2}+o_{p}\left(1\right), \end{array}$$

which, along with (A14), (A15) and (A25), implies

(A26) $$\begin{array}{cc} r_{k,\tau }= & \frac{1}{\sqrt{\left(\tau -\tau^{2}\right)\sigma_{a,\tau }^{2}}}\cdot \frac{1}{n}\sum_{t=k+1}^{n}\left\{\psi_{\tau }\left(\varepsilon_{t,\tau }\right)\left(\left|\varepsilon_{t-k,\tau }\right|-d_{1k}^{\prime }\Omega^{-1}\frac{z_{t}}{h_{t}}\right)\right.\\ & \left.+b_{\tau }f\left(b_{\tau }\right)\left(d_{2k}^{\prime }-d_{1k}^{\prime }\Omega^{-1}\Gamma \right)M^{-1}J^{⋆-1}\frac{1-\varepsilon_{t}^{⋆}}{2h_{t}^{⋆}\left(\theta_{0}^{⋆}\right)}\frac{\partial h_{t}^{⋆}\left(\theta_{0}^{⋆}\right)}{\partial \theta^{⋆}}\right\}+o_{p}\left(n^{-1/2}\right). \end{array}$$

As a consequence, for $R={\left(r_{1,\tau },\ldots ,r_{K,\tau }\right)}^{\prime }$, we have

(A27) $$\begin{array}{cc} R= & \frac{1}{\sqrt{\left(\tau -\tau^{2}\right)\sigma_{a,\tau }^{2}}}\cdot \frac{1}{n}\sum_{t=k+1}^{n}\left\{\psi_{\tau }\left(\varepsilon_{t,\tau }\right)\left(\epsilon_{t-1}-D_{1}\Omega^{-1}\frac{z_{t}}{h_{t}}\right)\right.\\ & \left.+b_{\tau }f\left(b_{\tau }\right)\left(D_{2}-D_{1}\Omega^{-1}\Gamma \right)M^{-1}J^{⋆-1}\frac{1-\varepsilon_{t}^{⋆}}{2h_{t}^{⋆}\left(\theta_{0}^{⋆}\right)}\frac{\partial h_{t}^{⋆}\left(\theta_{0}^{⋆}\right)}{\partial \theta^{⋆}}\right\}+o_{p}\left(n^{-1/2}\right), \end{array}$$

in which $\epsilon_{t-1}={\left(\left|\varepsilon_{t-1,\tau }\right|,\ldots ,\left|\varepsilon_{t-K,\tau }\right|\right)}^{\prime }$ and $D_{i}={\left(d_{i1},\ldots ,d_{iK}\right)}^{\prime }$ for i=1 and 2. Therefore, the desired result follows from the central limit theorem via the Cramér-Wold technique. □

**Proof of** **Theorem 4.** By an argument similar to that used to derive (A22), we have

(A28) $$\begin{array}{cc} & \frac{1}{\sqrt{n}}\sum_{t=k+1}^{n}\omega_{t}\psi_{\tau }\left({\hat{\varepsilon }}_{t,\tau }^{*}\right)\left|{\hat{\varepsilon }}_{t-k,\tau }^{*}\right|\\ =\; & \frac{1}{\sqrt{n}}\sum_{t=k+1}^{n}\omega_{t}\psi_{\tau }\left(\varepsilon_{t,\tau }\right)\left|\varepsilon_{t-k,\tau }\right|+\sum_{t=k+1}^{n}E_{1nt}^{*}+\sum_{t=k+1}^{n}E_{2nt}^{*}+\sum_{t=k+1}^{n}E_{3nt}^{*}, \end{array}$$

where

$$E_{1nt}^{*}=n^{-1/2}\omega_{t}\left[\psi_{\tau }\left({\hat{\varepsilon }}_{t,\tau }^{*}\right)-\psi_{\tau }\left(\varepsilon_{t,\tau }\right)\right]\left|\varepsilon_{t-k,\tau }\right|,$$

$$E_{2nt}^{*}=n^{-1/2}\omega_{t}\psi_{\tau }\left(\varepsilon_{t,\tau }\right)\left(\left|{\hat{\varepsilon }}_{t-k,\tau }^{*}\right|-\left|\varepsilon_{t-k,\tau }\right|\right),$$

$$E_{3nt}^{*}=n^{-1/2}\omega_{t}\left[\psi_{\tau }\left({\hat{\varepsilon }}_{t,\tau }^{*}\right)-\psi_{\tau }\left(\varepsilon_{t,\tau }\right)\right]\left(\left({\hat{\varepsilon }}_{t-k,\tau }^{*}\left|-\right|\varepsilon_{t-k,\tau }|\right)\right.$$

From (A18) and (A21), it is easy to know that

$$\sqrt{n}\left({\hat{\theta }}_{n}^{*}-\theta_{0}\right)=O_{p}\left(1\right),\;\;\sqrt{n}\left({\hat{\theta }}_{\tau n}^{*}-\theta_{\tau 0}\right)=O_{p}\left(1\right).$$

As a result, using methods similar to those in Zhang et al. [22], it can be shown that

$$\begin{array}{c} \sum_{t=k+1}^{n}E_{1nt}^{*}=-f\left(b_{\tau }\right)\left[d_{1k}^{\prime }\sqrt{n}\left({\hat{\theta }}_{\tau n}^{*}-\theta_{\tau 0}\right)+b_{\tau }d_{2k}^{\prime }\sqrt{n}\left({\hat{\theta }}_{n}^{*}-\theta_{0}\right)\right]+o_{p}\left(1\right), \end{array}$$

and

$$\begin{array}{c} \sum_{t=k+1}^{n}E_{\mathrm{int}\;}^{*}=o_{p}^{*}\left(1\right),\;i=2,3, \end{array}$$

in which $d_{1k}=E\left(h_{t}^{-1}\left|\varepsilon_{t-k,\tau }\right|z_{t}\right)$ and $d_{2k}=E(h_{t}^{-1}|\varepsilon_{t-k,\tau }\left|\sum_{j=1}^{p}\beta_{0j}\partial h_{t-j}\left(\theta_{0}\right)/\partial \theta \right)$. Consequently, together with (A25) and (A28), it follows that

$$\begin{array}{cc} & \frac{1}{\sqrt{n}}\sum_{t=k+1}^{n}\omega_{t}\psi_{\tau }\left({\hat{\varepsilon }}_{t,\tau }^{*}\right)\left|{\hat{\varepsilon }}_{t-k,\tau }^{*}\right|-\frac{1}{\sqrt{n}}\sum_{t=k+1}^{n}\psi_{\tau }\left({\hat{\varepsilon }}_{t,\tau }\right)\left|{\hat{\varepsilon }}_{t-k,\tau }\right|\\ =\; & \frac{1}{\sqrt{n}}\sum_{t=k+1}^{n}\left(\omega_{t}-1\right)\psi_{\tau }\left(\varepsilon_{t,\tau }\right)\left|\varepsilon_{t-k,\tau }\right|\\ & \;-f\left(b_{\tau }\right)\left[d_{1k}^{\prime }\sqrt{n}\left({\hat{\theta }}_{\tau n}^{*}-{\hat{\theta }}_{\tau n}^{⋆}\right)+b_{\tau }d_{2k}^{\prime }\sqrt{n}\left({\hat{\theta }}_{n}^{*}-{\hat{\theta }}_{n}^{⋆}\right)\right]+o_{p}\left(1\right), \end{array}$$

and hence it holds that

$$\begin{array}{cc} R^{*}-R= & \frac{1}{\sqrt{\left(\tau -\tau^{2}\right)\sigma_{a,\tau }^{2}}}\cdot \frac{1}{n}\sum_{t=k+1}^{n}\left(\omega_{t}-1\right)\left\{\psi_{\tau }\left(\varepsilon_{t,\tau }\right)\left(\epsilon_{t-1}-D_{1}\Omega^{-1}\frac{z_{t}}{h_{t}}\right)\right.\\ & \left.+b_{\tau }f\left(b_{\tau }\right)\left(D_{2}-D_{1}\Omega^{-1}\Gamma \right)M^{-1}J^{⋆-1}\frac{1-\varepsilon_{t}^{⋆}}{2h_{t}^{⋆}\left(\theta_{0}^{⋆}\right)}\frac{\partial h_{t}^{⋆}\left(\theta_{0}^{⋆}\right)}{\partial \theta^{⋆}}\right\}+o_{p}\left(n^{-1/2}\right), \end{array}$$

in which $\epsilon_{t-1}={\left(\left|\varepsilon_{t-1,\tau }\right|,\ldots ,\left|\varepsilon_{t-K,\tau }\right|\right)}^{\prime }$, and $D_{i}={\left(d_{i1},\ldots ,d_{iK}\right)}^{\prime }$ for i=1,2. The proof is therefore completed by invoking the Lindeberg central limit theorem together with the Cramér-Wold device. □
